# Strategies of Eradicating Glioma Cells: A Multi-Scale Mathematical Model with MiR-451-AMPK-mTOR Control

**DOI:** 10.1371/journal.pone.0114370

**Published:** 2015-01-28

**Authors:** Yangjin Kim, Gibin Powathil, Hyunji Kang, Dumitru Trucu, Hyeongi Kim, Sean Lawler, Mark Chaplain

**Affiliations:** 1 Department of Mathematics, Konkuk University, Seoul, 143-701, Republic of Korea; 2 Department of Mathematics, Ohio State University, Columbus, OH 43210, USA; 3 Division of Mathematics, University of Dundee, Dundee, UK; 4 Department of Physics, Konkuk University, Seoul, 143-701, Republic of Korea; 5 Department of Neurosurgery, Brigham and Women’s Hospital, Harvard Medical School, Boston MA 02115, USA; 6 Department of Mathematics, Swansea University, Swansea, UK; University of Pécs Medical School, HUNGARY

## Abstract

The cellular dispersion and therapeutic control of glioblastoma, the most aggressive type of primary brain cancer, depends critically on the migration patterns after surgery and intracellular responses of the individual cancer cells in response to external biochemical and biomechanical cues in the microenvironment. Recent studies have shown that a particular microRNA, miR-451, regulates downstream molecules including AMPK and mTOR to determine the balance between rapid proliferation and invasion in response to metabolic stress in the harsh tumor microenvironment. Surgical removal of main tumor is inevitably followed by recurrence of the tumor due to inaccessibility of dispersed tumor cells in normal brain tissue. In order to address this multi-scale nature of glioblastoma proliferation and invasion and its response to conventional treatment, we propose a hybrid model of glioblastoma that analyses spatio-temporal dynamics at the cellular level, linking individual tumor cells with the macroscopic behaviour of cell organization and the microenvironment, and with the intracellular dynamics of miR-451-AMPK-mTOR signaling within a tumour cell. The model identifies a key mechanism underlying the molecular switches between proliferative phase and migratory phase in response to metabolic stress and biophysical interaction between cells in response to fluctuating glucose levels in the presence of blood vessels (BVs). The model predicts that cell migration, therefore efficacy of the treatment, not only depends on oxygen and glucose availability but also on the relative balance between random motility and strength of chemoattractants. Effective control of growing cells near BV sites in addition to relocalization of *invisible* migratory cells back to the resection site was suggested as a way of eradicating these migratory cells.

## Introduction

Glioblastoma multiforme (GBM) is the most aggressive form of primary brain tumor with a median survival time of approximately 15 months from the time of diagnosis [1–3]. GBM is characterized by rapid proliferation and aggressive invasion into surrounding normal brain tissue, which leads to inevitable recurrence after surgical resection of the primary tumor site [4]. Surgery is the primary treatment method, followed by radiotherapy and chemotherapy. These approaches do not affect invasive GBM cells, which escape surgery and are protected behind the blood-brain barrier (BBB) and escape chemotherapy and many other cancer drugs. Innovative therapeutic approaches to target these invasive cells are needed in order to improve clinical outcome [5].

In the tumor microenvironment GBM cells encounter many challenges including hypoxia (lack of oxygen), acidity, and limited nutrient availability. To maintain rapid growth, tumor cells need to adapt to these biochemical changes in the harsh microenvironment [6]. In order to sustain their rapid growth, cancerous cells modify their metabolic activity by increasing glycolysis even in the presence of oxygen. This process requires high levels of glucose uptake and is known as the *Warburg effect* [7, 8]. In normal differentiated cells oxidative phosphorylation via the tricarboxylic acid (TCA), or Krebs cycle is the major energy producing mechanism. While differentiated cells favor this mode of metabolism which is very efficient in terms of ATP production, tumor cells adopt the seemingly inefficient process of aerobic glycolysis [9] due to production of lactic acid and consumption of large amounts of glucose [8]. Aerobic glycolysis [10] may give cancer cells the advantage of not having to depend on oxygen for energy especially in the hostile (hypoxic) tumor microenvironment, leading to longer survival [8, 10]. In order to survive periods of unfavorable metabolic stress and ensure an adequate nutrient supply as tumor mass accumulates, cancer cells develop strategies of metabolic adaptation [11], angiogenesis and migration [6]. Glioma cells are exposed to a challenging microenvironment where glucose levels may fluctuate due to heterogeneous biochemical and biophysical conditions. Therefore, adequate cellular responses to glucose withdrawal are critical for glioma cell survival in the harsh microenvironment. Under metabolic stress, cancer cells activate the 5′-adenosine monophosphate activated protein kinase (AMPK) pathway, the master cellular sensor of energy availability [12]. This way they enhance glucose uptake and to conserve energy [12], avoiding cell death.

**Figure 1:**
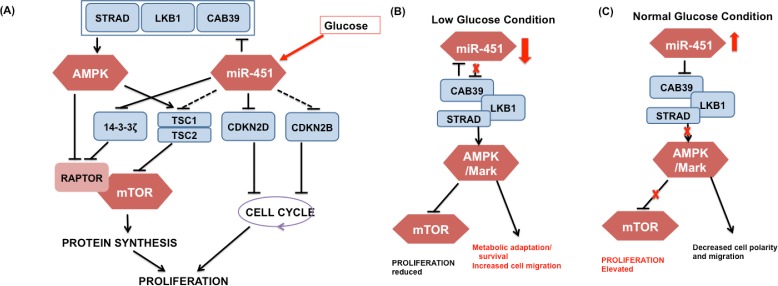
Signaling pathways in regulation of cell migration and proliferation in response to glucose. (A) Detailed multi-level action of miR-451 on proliferation-associated signaling pathways. Solid line = experimentally validated results, dashed line = putative results based on a prediction software. (B, C) Proposed role of the miR-451-AMPK-mTOR core control system in the regulation of cell proliferation and migration in response to high and low glucose levels. [6, 18] (CAB39/LKB1/AMPK/MARK) in the AMPK complex, which deactivates suppression of mTOR by the AMPK complex and leads to elevated proliferation and decreased cell polarity and migration. When the glucose level is low, down-regulation of miR-451 results in up-regulation of AMPK activity, which inhibits mTOR, thus leading to reduced proliferation, metabolic adaptation/survival, and increased cell polarity and migration through phosphorylation of MARKs. This also affects the cytoskeleton promoting cell migration for cell migration.

miRNAs are approximately 22 nucleotide single-stranded non-coding RNAs that are known to regulate gene expression [13]. Dysregulation of microRNA expression has been linked to oncogenic and tumor suppressor activities [14, 15] in several types of cancer, including GBM where altered miRNA expression contributes to tumorigenesis [16, 17]. For instance, miR-21 is highly expressed in GBM, and prevents apoptosis, and contributes to invasion through downregulating a number of tumor suppressor target genes. Godlewski *et al*. [6] have recently found that (i) under normal glucose conditions, over-expression of miR-451 leads to down-regulation of AMPK complex and elevated proliferation and decreased cell polarity/migration and (ii) glucose withdrawal induces down-regulation of miR-451 and up-regulation of AMPK activity, which in turn leads to increased cell polarity/migration and reduced cell proliferation. See Fig. 1 for a schematic summary of miR-451-AMPK-mTOR core control system.

We investigate the effect of fluctuations of oxygen and glucose from blood vessels on regulation of the core control system, miR-451-AMPK-mTOR, and explore the system behaviors in response to several different therapeutic interventions. We suggest the concept of localization *i.e.*, blocking invasiveness of glioma cells and attracting these cells back to the resection site, in order to eradicate invasive glioma cells. Our new model predicts the experimental observations *in vivo* [6, 18], in particular, localization of invasive cells near BV sites and over-expression of miR-451 in these cells [6]. Fluctuation in cell speeds during invasion processes for various random motility parameters were calculated and compared to experimental data. We propose that chemoattractant injection on the periphery of the tumor resection site immediately after surgery would bring most of those cells back to the resection site, making them detectable by Magnetic resonance imaging (MRI), and the follow-up surgery or radiation may improve survival rate of the patients by eradicating the remaining tumor cells. However, our study also predicts that S-G2-M- or G1-phase targeting chemo-drugs would also have to be administered in oder to kill proliferative cells near BVs.

## Materials and Methods

**Figure 2:**
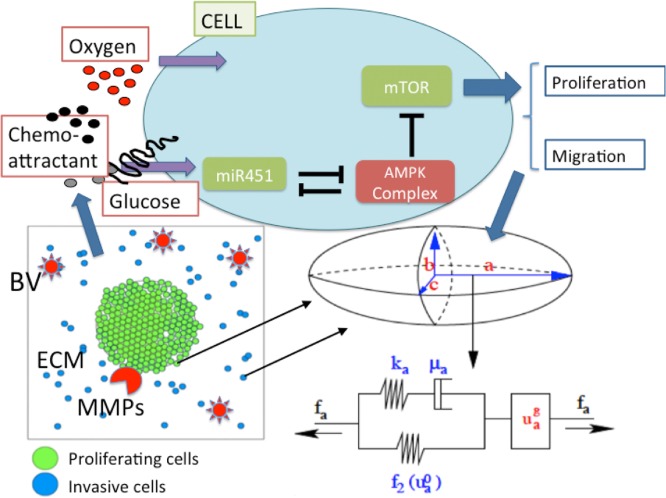
A schematic of the hybrid model. (Top) Intracellular dynamics of miR451-AMPK-mTOR, the core control system, in response to the diffusible molecules (glucose, oxygen, and chemoattractant) at a cell site. Those variables in the core system determine the cell fate, either proliferation or migration. (Bottom, Left) Model domain: Some proliferating tumor cells (green) on the surface of tumor mass are activated to become a motile one (blue) via miR-451-AMPK-mTOR regulation. Invasive cells secrete MMPs to degrade ECM and respond to chemotactic signals and biomechanical environmental factors such as blood vessels (BVs). (Bottom, Right) Changes in the length of the *a*-axis of a cell (the ellipsoid) under a given force (*f*_*a*_; arrow) consist of two components: (i) the passive change in the first component, a Maxwell element in parallel with a non-linear spring (ii) the change due to the growth ($u_{a}^{g}$). The growth component depends on the mTOR level and the force (*f*_*a*_). The mechanical and growth elements are the same along all axes. MMPs are secreted by invasive tumor cells and ECM components are degraded for invasion.

Our multiscale model contains several components and they are (1) intracellular miR-451-AMPK-mTOR pathway, (2) cell-based mechanical model, (3) reaction-diffusion model of extracellular biochemical players (oxygen, glucose, chemoattractants, Extracellular matrix (ECM), Matrix metalloproteinase (MMPs)). See Figure S4 in Supplementary File for schematic diagram of the hybrid model showing the appropriate scales involved. Consider brain tissue, Ω = [0, *L*] × [0, *L*], with a glioblastoma tumor initially occupying a sphere and blood vessels in the microenvironment (sources of glucose and oxygen). Tumor cells either proliferate or migrate under certain biochemical conditions of miR-451-AMPK-mTOR activation in the intracellular dynamics model in response to local concentrations of oxygen and glucose according to the reaction-diffusion model. While mechanical movement of the tumor cell is governed by the cell-based mechanical model, the migration direction is influenced by random motility and chemotaxis based on local concentrations of chemoattractants in the reaction-diffusion model. On the other hand, some dynamics in the reaction-diffusion model depend on individual-cell components; consumption of nutrients by cells and degradation of ECM by MMPs. Therefore, tumor cell growth and migration affect concentrations of molecules in the reaction-diffusion system and these changes are incorporated into the intracellular dynamics due to the spatial and temporal heterogeneity of oxygen and glucose levels. A schematic of the hybrid model is shown in Fig. 2.

### miR-451-AMPK-mTOR dynamics

**Figure 3:**
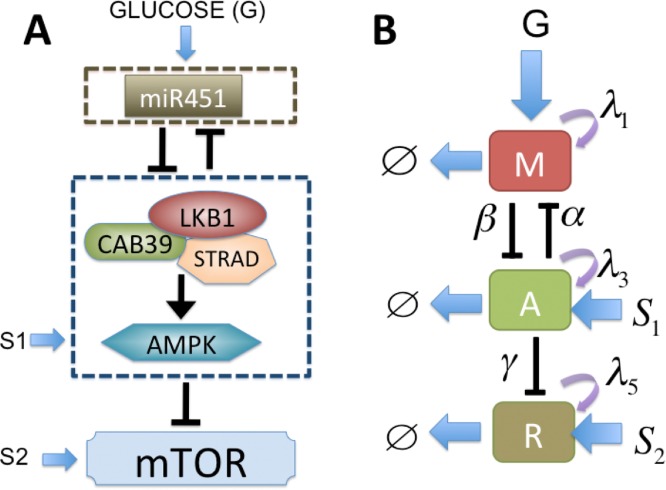
A simplified model of the network shown in Fig. 1. (A) Conceptual model of regulation of miR451, AMPK complex, and mTOR in GBM cell migration and proliferation [6]. (B) Cartoon model (extended from [41]): miR-451 level and activity of its target complex (CAB39/LKB1/AMPK), and mTOR levels were represented by ‘*M*’, ‘*A*’, and ‘*R*’, respectively.

We refer to the interactions represented by edges in Fig. 3B as the core miR-451-AMPK-mTOR control system. See Figure S1 in Supplementary File for the proposed role of miR-451 in the regulation of LKB1/AMPK-mTOR signaling in response to high and low glucose levels. By convention, the kinetic interpretation of arrows and hammerheads in the network represents induction (arrow) and inhibition (hammerhead). miR-451 level and activity of its target complex (CAB39/LKB1/AMPK), and mTOR levels are represented by ‘*M*’, ‘*A*’, and ‘*R*’, respectively. By simplifying the network as shown in Fig. 3A, we obtain the following dimensionless version of the model based on Fig. 3B

$$\frac{dM}{dt}=\lambda_{g}G+\frac{\lambda_{1}\lambda_{2}^{2}}{\lambda_{2}^{2}+\alpha A^{2}}-M,$$ (1)

$$\epsilon_{1}\frac{dA}{dt}=S_{1}+\frac{\lambda_{3}\lambda_{4}^{2}}{\lambda_{4}^{2}+\beta M^{2}}-A,$$ (2)

$$\epsilon_{2}\frac{dR}{dt}=S_{2}+\frac{\lambda_{5}\lambda_{6}^{2}}{\lambda_{6}^{2}+\gamma A^{2}}-R.$$ (3)

where *G* is the signaling pathways from glucose to miR-451, *S*_1_, *S*_2_ are the signalling pathways to AMPK complex and mTOR, respectively, λ_1_, λ_3_, λ_5_ are the autocatalytic enhancement parameters for miR-451, AMPK complex, mTOR, respectively and λ_2_, λ_4_, λ_6_ are the Hill-type inhibition saturation parameters from the counter part of miR-451, AMPK complex, mTOR, respectively, *α* is the inhibition strength of miR-451 by the AMPK complex, *β* is the inhibition strength of the AMPK complex by miR-451, and finally *γ* is the inhibition strength of the mTOR by the AMPK complex. The parameter *ϵ*_1_ is given by the ratio of the degradation rates of *M* and *A*, respectively. Similarly, the parameter *ϵ*_2_ is given by *μ*_1_/*μ*_3_, the ratio of the degradation rates of *M* and *R*, respectively.

The parameters for the equations (1)–(3) are given in Table 1 and are referred to as essential control parameters. See Supplementary materials for the derivation of the model based on a dimensional cartoon model in Figure S2 in Supplementary File.

**Table 1:** Parameters used in the intracellular miR-451-AMPK-mTOR system.

	Description	Value	Refs
λ_*g*_	glucose signaling rate	1.0	[41]
λ_1_	autocatalytic production rate of miR-451	4.0	[41]
λ_2_	Hill-type coefficient	1.0	[41]
*α*	Inhibition strength of miR-451 by AMPK complex	1.6	[41]
*th*_*M*_	Threshold of AMPK for proliferation/migration switch	2.0	Estimated
λ_3_	autocatalytic production rate of AMPK	4.0	[41]
λ_4_	Hill-type coefficient	1.0	[41]
*β*	Inhibition strength of AMPK complex by miR-451	1.0	[41]
*S*_1_	Signaling source of AMPK	0.2	[41]
*ϵ*_1_	Scaling factor (slow dynamics) of AMPK complex	0.02	[15, 41, 51, 52]
*th*_*A*_	Threshold of AMPK for proliferation/migration switch	2.0	Estimated
λ_5_	autocatalytic production rate of mTOR	4.0	Estimated
λ_6_	Hill-type coefficient of mTOR module	1.0	Estimated
*γ*	Inhibition strength of mTOR activity by AMPK	1.0	Estimated
*S*_2_	Signaling source of mTOR	1.2	Estimated
*ϵ*_2_	Scaling factor (slow dynamics) of mTOR	0.02	[15, 51, 52], estimated
*th*_*R*_	Threshold of AMPK for proliferation/migration switch	3.0	Estimated

### Mechanical effects on tumor growth: The cell-based component

Cell-mechanics is believed to play a large role in tumor growth and invasion. Mechanical stresses and the signaling pathways influence growth in a phenomenologically-specified manner in our model.

#### The forces acting on individual cells

The mechanical behavior of individual cells is based on the model developed by Dallon and Othmer [19] (hereafter the paper and model is denoted DO) and Kim *et al*. [20] (hereafter the paper and model is denoted KSO, a hybrid model). The basic scheme for cell division in the context of tumor growth was developed in the KSO model [20]. Further applications of the hybrid model to glioblastoma and breast cancer can be found in [21–23]. The new aspect that is needed in the present context is the core control system (miR-451-AMPK-mTOR) for cell proliferation and migration. The forces on a cell in the DO model include, (i) the traction forces exerted on neighboring cells or the substrate, (ii) the dynamic drag forces that arise as a moving cell forms and breaks adhesive bonds with neighboring cells, (iii) a static frictional force that exists when cells are rigidly attached to each other or to the substrate, and (iv) a reactive force due to forces exerted by other cells on it. The traction force on a cell *i* is denoted **T**_*i, j*_, wherein *j* = 0 denotes the substrate, and the reaction force to this is denoted **M**_*j, i*_. In the context of glioma migration regulated by miR-451-AMPK-mTOR, the specific form of active forces will be introduced Section below. The static force, which is denoted **S**_*j, i*_, is the binding force on the *i*th cell when bound to the *j*th. Since **S**_*i, j*_ = -**S**_*j, i*_, the cell-cell forces cancel on all but those cells attached to the substrate. A more detailed discussion of all forces involved can be found in [19, 20]. The total force on the *i*th cell is then summarized by

$$F_{i}=\sum_{j\in N_{i}^{a}}M_{j,i}+\sum_{j\in N_{i}^{a}}T_{j,i}+\sum_{j\in N_{i}^{d}}\mu_{ij}(v_{j}-v_{i})+\sum_{j\in N_{i}^{s}}S_{j,i}$$ (4)

where $N_{i}^{a}$ denotes the neighbors of *i*, including the substrate, upon which it can exert traction, $N_{i}^{d}$ is the set of cells (which includes substrate and extracellular matrix) that interact with *i* via a frictional force, and $N_{i}^{s}$ denotes the set of cells that statically bind to cell *i*.

#### Cell growth and the rheology of the cytoplasm

There are two different kinds of cells involved in the system: proliferative cells and motile cells. The cells are treated as oriented ellipsoids and their cytoplasm considered as an incompressible, viscoelastic solid. Based on previous work [19–21], the governing equations of the length of the *i*-th axis, *i* = **a**, **b**, **c**, of a cell are

$$u_{i}=u_{i}^{0}+u_{i}^{g},$$ (5)

$$\frac{du_{i}^{0}}{dt}=(\frac{k_{i}}{\mu_{i}}[f_{i}(t)+\bar{p}-f_{2}(u_{i}^{0})]+\frac{df_{i}}{dt})\times {\left(f_{2}^{\prime }(u_{i}^{0})+k_{i}\right)}^{-1},$$ (6)

where *u*_*i*_ is the change in the length of the *i*th axis, $u_{i}^{0}$ and $u_{i}^{g}$ are the changes in the length of the *i*th axis due to a change in the passive and growth element, respectively, *f*_*i*_ is the magnitude of the force applied at each end, *f*_2_ is the nonlinear spring force from the spring in parallel, *k*_*i*_ is the spring constant for the spring in the Maxwell element, *μ*_*i*_ is the viscous coefficient of the dashpot, $\bar{p}$ is the force due to pressure. See [19] for specific form of the function *f*_2_ and details of how these equations are established. It is assumed that the passive response is incompressible [20, 21], leading to the volume constraint for $u_{a}^{0}$, $u_{b}^{0}$, and $u_{c}^{0}$. Specific and detailed form of the growth term $\frac{du_{i}^{g}}{dt}$ is described in details in Supplementary File. The growth rate function for the *i*-th axis is given by

$$\frac{du_{i}^{g}}{dt}=f(\sigma )\cdot P(M,A,R,[G_{0}])$$ (7)

$$P(M,A,R,[G_{0}])=\{\begin{array}{ll} 1 & \text{if}\;M>th_{M},A<th_{A},R>th_{R},[G_{0}]=0\\ 0 & \text{otherwise} \end{array}$$ (8)

where *σ* is the force acting on the cell and *P* is a function of the levels of miR-451 (*M*), AMPK complex (*A*), and mTOR (*R*), and quiescent status ([*G*_0_]) in the cell cycle. Here, *th*_*M*_, *th*_*A*_, *th*_*R*_ are thresholds of miR-451, AMPK complex, and mTOR, respectively. The growth function *f*(*σ*) is defined so that cells do not grow if forces are too large, but can grow under sufficiently small tensile and compressive forces [20]. (See equation (13) and discussion in the Supplementary file for more detailed description of *f*(*σ*).) As a result growth is either on or off in the context of mechanical stress acting on the cell. Cell proliferation depends on levels of many intracellular variables that control internal biological clocks of cell proliferation and migration. In the present work, we assume that the core control system and conditions of *G*_0_ phase ([*G*_0_] = 0 or 1) in the cell cycle module also determine cell proliferation, *i.e.*, when the cell *i* is getting the right proliferation signal from the core control system (miR-451-AMPK-mTOR) and the cell is in the regular cell-cycle ([*G*_0_] = 0). Biochemical conditions for the appropriate proliferation signals are based on experimental study [6] where up-regulation of miR-451 and mTOR, and down-regulation of the AMPK complex lead to proliferation (*M* > *th*_*M*_, *A* < *th*_*A*_, *R* > *th*_*R*_) while down-regulation of miR-451 and mTOR and up-regulation of AMPK complex induce cell migration (*M* < *th*_*M*_, *A* > *th*_*A*_, *R* < *th*_*R*_). By setting appropriate thresholds *th*_*M*_, *th*_*A*_, *th*_*R*_, we will define a proliferative ($T_{p}$) and migrative ($T_{m}$) phases, respectively. (see equations (20)–(21) in Results section.) The quiescent status (*G*_0_-phase) is given either 1 or 0 when the cell gets quiescent cues from signaling pathways other than the miR-4510AMPK-mTOR signaling.

#### Active force and equations of motion

The active force is the force that a cell generates via complex biochemical and bio-mechanical processes in order to move. These processes include generation of traction forces on the adhesion sites [19], tension, and activation of the acto-myosin machinery [24], in particular myosin II for glioma cells infiltrating brain tissue [25]. In the original lattice-free cell-based models [19, 26], the traction force exerted by a cell in the interior of a cell aggregate is exerted on one of its neighboring cells. For a cell in contact with a flat 2-dimensional substrate, the ‘pulling’ process on the substrate gives rise to the component of the force in the plane of the substrate. If a cell is not in contact with the substrate, the entire force is obtained by pulling on the neighboring cell whose center is closest to the line of the desired motion. (See Dallon and Othmer [19] for more detail.) In this work, we do not consider the detailed acto-myosin machinery nor collective cell migration which are key players in a certain stage of many cancers [24]. Here, we have used a simplified description, in which we assume that the traction force for active cell migration is generated for only the cells (i) that are in contact with the substrate (*i.e.*, without physical constraints) and (ii) that receive the correct migratory signal from the miR-451-AMPK-mTOR control system. Therefore, the migratory cells do not depend on neighboring cells for active migration but they can transmit the traction force directly to the substrate in brain tissue without pulling its neighboring cells. The model can be extended to incorporate traction forces generated by all cells, but we consider only migratory individual cells due to their rather dispersal migration patterns in glioma invasion [27, 28].

The traction force $T_{i}^{a}$ for migratory cell *i* is given by

$$T_{i}^{a}=\phi (A)(\psi_{1}d_{r}+\psi_{2}\frac{\nabla G}{\sqrt{K_{G}+|\nabla G{|}^{2}}}+\psi_{3}\frac{\nabla C}{\sqrt{K_{C}+|\nabla C{|}^{2}}})\equiv \phi (A)T_{i,g}$$ (9)

where **d**_*r*_ is a unit vector of the moving direction from random motion, *G*, *C* are the concentrations of glucose and a chemoattractant, respectively (described in Section above), *ψ*_1_, *ψ*_2_, *ψ*_3_ are scaling factors of weight distribution favoring random motion, glucose and other chemoattractants, respectively (*ψ*_1_, *ψ*_2_, *ψ*_3_ ∊ [0, 1]; *ψ*_1_+*ψ*_2_+*ψ*_3_ = 1), *A* is the level of AMPK complex at the *i*th cell site. We allow a small randomness in the magnitude of traction force. Then, the indicator function *ϕ*(*A*) is given by

$$\begin{array}{l} \phi (A)=\{\begin{array}{ll} F_{0}\phi_{r} & \;\text{if}\;A_{i}>th_{A}\;\text{and the cell does not have physical constraints}\\ 0 & \text{otherwise}, \end{array} \end{array}$$ (10)

where *F*_0_ is the basal magnitude of the traction force ($0\leq |T_{i}^{a}|\leq F_{0}$) and *ϕ*_*r*_ is a random number (*ϕ*_*r*_ ∊ [0.8, 1.2]). Therefore, the traction force is completely turned off for cells in the proliferative phase (*M* > *th*_*A*_, *A* < *th*_*A*_, *R* > *th*_*R*_) or cells under physical constraints. If there are other cells 90° in any direction (**T**_*i, g*_) of motion of cell *i*, the cell *i* is defined to be under physical constraints. For a more precise algorithm, let *j*, the cell index for the cell whose center is in the closest direction to the direction of **T**_*i, g*_, be given by $j=\{k:max_{k\in N_{i}^{a}}\frac{x_{k}-x_{i}}{\left|x_{k}-x_{i}\right|}\cdot {\hat{T}}_{i}\}$ where **x**_*n*_ is the location of the center of the *n*-th cell, $N_{i}^{a}=\{k:0<|x_{k}-x_{i}|\leq d_{n}\}$, the neighboring cells of the *i*-th cell, and ${\hat{T}}_{i}=\frac{T_{i,g}}{\left|T_{i,g}\right|}$. The cell *i* is defined to be under physical constraints if *j* > 0, *i.e.*, when another cell is blocking the way in the migration direction ${\hat{T}}_{i}$. For example, when a cell is completely surrounded by neighboring cells, such as a cell within the growing tumor core, the traction force is turned off. From the formulation in the equation (9), it would be turned off when gradients of glucose (∇*G*) and chemoattractants (∇*C*) are zero in the absence of random motility (*ψ*_1_ = 0), *i.e.*, no traction force is generated in the absence of chemotactic signals.

In view of these assumptions above, the force balance on migratory cells involves the reaction $T_{i}^{a,*}=-T_{i}^{a}$ to the traction force $T_{i}^{a}$, adhesion forces between cells (**A**_*i, j*_), the drag due to the fluid acting on the glioma cell, internal forces (**R**_*j, i*_), and the passive reactive force due to cell-substrate ($R_{0,i}^{*}$) and cell-cell deformation ($R_{j,i}^{*}$). In summary, by using equations (4)–(10) and neglecting acceleration due to slow movement of cells, Newton’s law for the *i*th cell reduces to

$$\begin{array}{c} A_{if}\mu_{f}v_{i}+A_{is}\mu_{s}v_{i}+\mu_{\mathrm{cell}}\sum_{j\in N_{i}}A_{ij}(v_{i}-v_{j})+\\ +\frac{A}{6\pi r_{ib}}\left(T_{i}^{a,*}+R_{0,i}^{*}+\sum_{j\in N_{i}}A_{i,j}+\sum_{j\in N_{i}}R_{j,i}+\sum_{j\in N_{i}}R_{j,i}^{*}\right)=0, \end{array}$$ (11)

where **v**_*i*_ is the velocity of cell *i*, $N_{i}$ is the neighborhood of cell *i*, *μ*_*cell*_ (resp., *μ*_*s*_, *μ*_*f*_) is the degree of adhesiveness between the cells (resp., between the substrate and the cells, and the fluid viscosity), *r*_*ib*_ = *u*_*b*_+*b*_0_, and *A*_*ij*_ = *A*_*ij*_(*t*), *A*_*if*_ = *A*_*if*_(*t*), *A*_*is*_ are the areas of contact regions between cell *i* and cell *j*, cell *i* and the interstitial fluid or matrix, and cell *i* and the substrate at time *t* respectively, *A* = *A*(*t*) is the total area of an undeformed cell. The solution of this equation (11) provides a trajectory of cell *i*. For more details see Dallon and Othmer [19]. Parameters in the cell-based component are listed in Table 2.

**Table 2:** Parameters for the cell-based component of the model. TW = this work. *dimensionless value.

Parameter	Description	Value	Refs.
Adhesion parameters			
*μ*_*cell*_	cell-cell adhesiveness	27.0 dyn s/cm	[19]
*μ*_*s*_	cell-substrate adhesiveness	27.0 dyn s/cm	[19]
*μ*_*f*_	the fluid viscosity	2.7 dyn s/cm	[19]
Rheological parameters			
*c* ^+^	Growth function parameter	1.016089 × 10^–7^ mm/(min.nN)	[20], TW
*σ* ^+^	Growth function parameter	800 nN	[20]
*σ* ^–^	Growth function parameter	-4 nN	[20]
*k*_*a*_	Standard solid parameter in cell	163.8 dyn/cm	[19, 20]
*k*_2_	Standard solid parameter in cell	147.5 dyn/cm,	[19, 20]
*μ*_*a*_	Standard solid parameter in cell	123 dyn min/cm	[19, 20]
Active force parameters			
*ψ*_1_	Weight for random motility (*ψ*_1_+*ψ*_2_+*ψ*_3_ = 1)	0–1.0	TW
*ψ*_2_	Weight for glucose gradient (*ψ*_1_+*ψ*_2_+*ψ*_3_ = 1)	0–1.0	TW
*ψ*_3_	Weight for chemoattractant gradient (*ψ*_1_+*ψ*_2_+*ψ*_3_ = 1)	0–1.0	TW
*F*_0_	Maximal active force magnitude	64 *nN*	[19], TW
*ϕ*_*r*_	Random factor for basal active force	0.8–1.2	[19], TW
*K*_*G*_	Active force scaler for the glucose gradient	1.0*	TW
*K*_*C*_	Active force scaler for the chemoattractant gradient	1.0*	TW

### Dynamics of biochemical players

The macroscopic dynamics of concentrations of relevant biochemical players (oxygen, glucose, chemoattractants, ECM, MMPs, and chemotherapeutic) is modelled using a suitable partial differential equation incorporating vessels as sources within the simulation domain. Let *K*(**x**, *t*), *G*(**x**, *t*), *C*(**x**, *t*), *ρ*(**x**, *t*), and *P*(**x**, *t*) denote the concentration of oxygen, glucose, chemoattractants, ECM, and MMPs at position x at time *t*, respectively. Their rate of change can be expressed as

$$\frac{\partial K}{\partial t}=\nabla \cdot (D_{K}(x)\nabla K)+r_{K}I_{B}(x)-l_{c}^{K}I_{C_{T}}(x)-\mu_{K}K$$ (12)

$$\frac{\partial G}{\partial t}=\nabla \cdot (D_{G}(x)\nabla G)+r_{G}I_{B}(x)-l_{c}^{G}I_{C_{T}}(x)-\mu_{G}G$$ (13)

$$\frac{\partial C}{\partial t}=\nabla \cdot (D_{C}(x)\nabla C)+\sum_{j=1}^{N_{C}}l_{in}^{C}I_{[t_{j}^{C},t_{j}^{C}+\tau_{d}^{C}]\times \Omega_{R}}-\mu_{C}C\;\text{in}\;\Omega ,$$ (14)

$$\frac{\partial \rho }{\partial t}=-l_{1}P\rho +l_{2}\rho \left(1-\frac{\rho }{\rho_{*}}\right)\;\text{in}\;\Omega ,$$ (15)

$$\frac{\partial P}{\partial t}=\nabla (D_{P}(x)\nabla P)+l_{3}\;I_{C_{m}}(x)-\mu_{P}P\;\text{in}\;\Omega ,$$ (16)

where *D_K_*(**x**), *D_G_*(**x**), *D_C_*(**x**), *D_P_*(**x**) are the diffusion coefficients of oxygen, glucose, chemoattractant, and MMPs, respectively, *μ*_*C*_, *μ*_*P*_ are the natural decay rates of chemoattractant and MMPs, respectively. The third terms in the equations (12)–(13) are a function describing the consumption of oxygen and glucose by tumour cells at rates $l_{c}^{K},l_{c}^{G}$, respectively. The fourth terms in the equations (12)–(13) represent the consumption of oxygen and glucose by other cells in the brain tissue at rates *μ*_*K*_, *μ*_*G*_. Here, *I*_*C*_*T*__(⋅) is an indicator function on the tumor sites *C*_*T*_ (including both proliferative and migratory cells)

$$I_{C_{T}}(x)=\{\begin{array}{ll} 1 & \text{tumor cells}\\ 0 & \text{otherwise}. \end{array}$$ (17)

Similarly, *I*_*B*_(⋅) is an indicator function on the set *B* that represents the blood vessel:

$$I_{B}(x)=\{\begin{array}{ll} 1 & \text{blood vessels}\\ 0 & \text{otherwise}. \end{array}$$ (18)

Thus the terms *r*_*K*_ *I*_*B*_(⋅) and *r*_*G*_ *I*_*B*_(⋅) in the equations (12)–(13) describe the supply of oxygen and glucose via blood vessel at rates *r*_*K*_ and *r*_*G*_, respectively. We assume that the oxygen and glucose are supplied through the pre-existing blood vessels, and then diffuse throughout the tissue feeding the cells within its diffusion limit. As the tumour cells grow, some of the cells within the tumour mass will be starved of oxygen and glucose which in turn affect their intracellular processes, enabling them to adapt to the changing microenvironment. These changes due to spatial and temporal heterogeneity of oxygen and glucose levels is incorporated into the model using the intracellular dynamics described in the previous section. The second term in the equation (14) represents injection of chemoattractants at a rate $l_{in}^{C}$ on a set Ω_*R*_ near the resection cavity after conventional surgery. These injections are administered over the time intervals $\left[\tau_{j}^{C},\tau_{j}^{C}+\tau_{d}^{C}\right]$ ($j=1,\ldots ,N_{C};\;\tau_{1}^{C}<\tau_{2}^{C}<\ldots <\tau_{N_{C}}^{C}$) where $\tau_{d}^{C}$ is an equal duration of injection and *N*_*C*_ is the total number of injections. Due to its injection location, this injection in addition to the diffusion effect and natural decay would create a gradient of chemoattractants, which would serve as a localization of migratory glioma cells back to the area near the periphery of the resection cavity for second surgery or other anti-tumor treatments. Even though the ECM provides a structural support for the guidance of cell migration, high density of ECM also blocks cell migration and a migratory cell need to produce proteinases such as MMPs in order to infiltrate the brain tissue. The first and second terms in the equation (15) represent the degradation of the ECM by MMPs secreted by invasive tumor cells at a rate *l*_1_ and release/reconstruction of the ECM at a rate *l*_2_, respectively. MMPs are secreted by *invasive* tumor cells at a rate *l*_3_ in the equation (16) when cell motility is necessary, therefore, its concentration is localized at the location (*I*_*C*_*m*__) of glioma cells in the migratory phase (*M* < *th*_*M*_, *A* > *th*_*A*_, *R* < *th*_*R*_). Here, *I*_*C*_*m*__(⋅) is an indicator function on the set *C*_*m*_ that represents *invasive* cell sites:

$$I_{C_{m}}(x)=\{\begin{array}{ll} 1 & \;\text{if}\;(M,A,R)\;\text{at}\;\text{cell}\;i\in T_{m}\\ 0 & \text{otherwise}. \end{array}$$ (19)

where $T_{m}$ is the migratory phase, which will be defined precisely later. (see equations (20)–(21) in Results section.) We assume no flux (Neumann) boundary conditions for the system (12)–(16). Parameters in the cell-based component are listed in Table 3.

**Table 3:** Parameters that are used in the reaction-diffusion equations.

	Description	Value	Refs
Diffusion Coefficients			
*D*_*K*_	Oxygen	2.0 × 10^–5^ *cm* ^2^/*s*	[42–44]
*D*_*G*_	Glucose	6.7 × 10^–7^ *cm* ^2^/*s*	[28, 45, 46]
*D*_*C*_	Chemoattractant (EGF)	1.66 × 10^–6^ *cm* ^2^/*s*	[47]
*D*_*P*_	MMPs	8.0 × 10^–9^ *cm* ^2^/*s*	[48]
*D*_*D*_	Chemo-drug (CYC202)	6.9 × 10^–6^ *cm* ^2^/*s*	[49, 50]
Production Rates			
*r*_*K*_	Oxygen supply rate from blood	6.35 × 10^–4^ *g*/(*cm* ^3^.*s*)	TW
*r*_*G*_	Glucose supply rate from blood	1.4 × 10^–3^ *g*/(*cm* ^3^.*s*)	TW
$l_{in}^{C}$	Chemoattractant injection rate	2.68 × 10^–9^ *g*/(*cm* ^3^.*s*)	TW
*l*_2_	ECM reconstruction/remodelling rate	5.6 × 10^–3^ *s* ^–1^	TW
*l*_3_	MMP production rate	(2.32 × 10^–11^- 3.15 × 10^–9^) *gcm* ^–3^ *s* ^–1^	[23, 28, 53], TW
ρ_*_	ECM carrying capacity	= ρ_*_	[28, 54, 55]
Decay/Consumption Rates			
$l_{c}^{K}$	Oxygen consumption rate by tumor	0.8 *pg*/*cell*/*min*	TW
$l_{c}^{G}$	Glucose consumption rate by tumor	0.8 *pg*/*cell*/*min*	[56, 57], TW
*l*_1_	ECM degradation rate by MMPs	3.0 × 10^4^ *cm* ^3^ *g* ^–1^ *s* ^–1^	[55], TW
*μ*_*K*_	Removal rate of oxygen in brain tissue	2.0 × 10^–5^ *s* ^–1^	[58], TW
*μ*_*G*_	Removal rate of glucose in brain tissue	0.0034 *min* ^–1^	TW
*μ*_*C*_	Decay rate of chemoattractant (EGF)	8.02 × 10^–6^ *s* ^–1^	[59]
*μ*_*P*_	Decay rate of MMPs	5.0 × 10^–5^ *s* ^–1^	[54, 55]
*μ*_*D*_	Decay rate of chemo-drugs (CYC202)	1.849 *h* ^–1^	[49, 50]

## Results

In this Section, we present an analysis of the hybrid model and predictions for therapeutic strategies for eliminating invasive glioma cells. We will initially analyze the core control system (miR-451-AMPK-mTOR), one of the modules in the hybrid model, in the context of control of cell proliferation and migration. Then, we will incorporate the core control system into the full hybrid model and analyze the dynamics of the model system and behavior of proliferative and migratory cells under the control of the miR-451-AMPK-mTOR module in response to bio-mechanical/chemical factors such as physical constraints, fluctuating glucose levels and BVs after conventional surgery. We show that our simulation results are in good agreement with experimental observations [6, 18]. We then explore the growth/invasion patterns with differing random cell motility (*ψ*_1_) and BV density, and investigate the efficacy of conventional chemotherapy after surgery using the hybrid model. Finally, we propose a localization strategy as a way of eradicating the *invisible* migratory cancer cells in combination with chemotherapy after surgery, which can be an alternative to conventional surgery-chemo/radio therapy treatment options used to prevent the recurrence of glioma after surgery. We also show that cell speed and changes in migration direction are consistent with experimental observation.

### Dynamics of the core control (miR-451-AMPK-mTOR) system

We recall that low levels of miR-451 (up-regulated AMPK complex and down-regulated mTOR) induce reduced cell proliferation and increased cell motility while over expression of miR-451 (down-regulation of AMPK complex and up-regulation of mTOR) leads to elevated cell proliferation and reduced migration in experiments [6, 18]. In order to take into account the effect of glucose conditions in our model on phenotypic changes (proliferative and migratory cells), we first test how the glucose level (*G*) affects the levels of key players (*M*, *A*, *R*) in our core control system.

**Figure 4:**
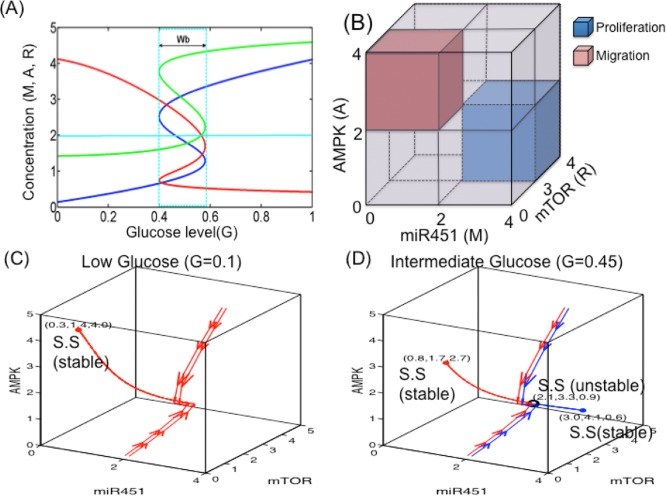
Bifurcation curve of the miR-451-AMPK-mTOR module. (A) High and low glucose levels (*G*) provide an on-off switch of miR-451 over-expression and determine the dichotomous behavior: cell proliferation or migration [41]. Y-axis = steady state (SS) of miR-451, AMPK, and mTOR. $W_{b}=[b_{m}^{w},b_{M}^{w}]=a$ window of bi-stabilty. (B) Characterization of proliferation and migration of glioma cells in miR-451/mTOR-AMPK domain. The proliferative region (blue box) is defined as the region where the miR-451 level is above a threshold, *th*_*M*_ (*M* > *th*_*M*_), the AMPK level is below a threshold, *th*_*A*_ (*A* > *th*_*A*_), and the mTOR level is above a threshold, *th*_*R*_ (*R* > *th*_*R*_), while the levels of miR-451, AMPK, and mTOR in the migratory region (red box) satisfies *M* < *th*_*M*_, *A* > *th*_*A*_, *R* < *th*_*R*_. We set *th*_*M*_ = 2.0, *th*_*A*_ = 2.0, *th*_*R*_ = 3.0. (C, D) Dynamics of the core control system in response to low (*G* = 0.1 in (C)) and intermediate (*G* = 0.45 in (D)) glucose levels. When *G* = 0.1, there is only one stable steady state (red dot, (*M* ^*s*^, *R* ^*s*^, *A* ^*s*^) = (0.3, 1.4, 4.0)) in the migratory region while, for *G* = 0.45, there are three steady states: two stable steady states (red dot ((*M* ^*s*^, *R* ^*s*^, *A* ^*s*^) = (1.8, 1.7, 2.7)) in the migratory region and blue dot ((*M* ^*s*^, *R* ^*s*^, *A* ^*s*^) = (3.0, 4.1, 0.6)) in proliferative regions) and one unstable steady state (black circle (((*M* ^*s*^, *R* ^*s*^, *A* ^*s*^) = (2.1, 3.3, 0.9)) in the middle). Red and blue curves indicate the trajectories of solutions for two pairs of very close initial conditions: (*M*, *A*, *R*)(0) = (2.1, 4.0, 5.0) and (2.2, 4.0, 5.0); (*M*, *A*, *R*)(0) = (2.1, 0.1, 0.1), and (2.2, 0.1, 0.1).

When the core miR-451-AMPK-mTOR system (1)–(3) is in equilibrium, we can solve miR-451 levels (steady state (S.S) value *M* ^*s*^) as a function of the extracellular glucose level (*G*). In a similar fashion, we can also obtain the bifurcation curve of steady state values of AMPK activity (*A* ^*s*^), and mTOR levels (*R* ^*s*^) w.r.t. external glucose signal (*G*). Fig. 4A shows the graphs *M* = *M*(*G*) (blue), *A* = *A*(*G*) (red), *R* = *R*(*G*) (green) as a *S*-shaped curve (hysteresis) with reversed direction of the *A*-curve. While the upper and lower branches of those curves are stable, the middle branch is unstable. Under glucose withdrawal conditions, the system (1)–(3) travels along the lower branch (*M* low, *A* high, *R* low) of the miR-451 curve and the cells are in the migratory phase. The cell continues to migrate as *G* is increased until it reaches the right knee point of the bifurcation curve (∼0.6). Around this point, both miR-451 and mTOR levels jump to the upper branch, with elevated levels of miR-451 and mTOR and down-regulated AMPK, and the cells are put in the proliferative phase (migration switch is turned off). Theofore, the effect of glucose is history dependent. The size of the bi-stability window ($W_{b}=[b_{m}^{w},b_{M}^{w}]$) depends on other parameters and may even disappear under the perturbation of some parameters. By taking the thresholds, *th*_*M*_ (= 2.0) of miR-451 levels, *th*_*A*_ (= 2.0) of AMPK complex, and *th*_*R*_ (= 3.0) of mTOR, we shall define the migratory region $T_{m}$ (red box in Fig. 4B) by

$$T_{m}=\{(M,A,R)\in R^{3}:M<th_{M},\;A>th_{A},\;R<th_{R}\}$$ (20)

and the proliferative region $T_{p}$ (blue boxes in Fig. 4) by

$$T_{p}=\{(M,A,R)\in R^{3}:M>th_{M},\;A<th_{A},\;R>th_{R}\}.$$ (21)

For an illustration of the dynamics, Figs. 4C, 4D show the dynamics of the core control system in response to low (*G* = 0.1; Fig. 4C) and intermediate (*G* = 0.45; Fig. 4D) glucose levels. For the low glucose level (*G* = 0.1; Fig. 4C), there is only one stable steady state (red dot; (*M* ^*s*^, *R* ^*s*^, *A* ^*s*^) = (0.3, 1.4, 4.0)) in the migratory region $T_{m}$ where miR-451 (*M*) and mTOR (*R*) expressions are low but AMPK activity (*A*) is high. On the other hand, for high glucose levels (*G* > 0.6), there is only one stable steady state in a region $T_{p}$ where miR-451 (*M*) and mTOR (*R*) expressions are high but AMPK activity (*A*) is low (data not shown; *cf*. Fig. 4A). For an intermediate glucose level in the bi-stability window *G* = 0.45 ∊ *W*_*b*_ in Fig. 4D), there are three steady states: one stable S.S. (red dot; (*M* ^*s*^, *R* ^*s*^, *A* ^*s*^) = (1.8, 1.7, 2.7)) in the high AMPK activity region $T_{m}$, another stable S.S (blue dot; (*M* ^*s*^, *R* ^*s*^, *A* ^*s*^) = (3.0, 4.1, 0.6)) in the high miR-451 activity region $T_{p}$), and one unstable steady state in the middle (black circle; (*M* ^*s*^, *R* ^*s*^, *A* ^*s*^) = (2.1, 3.3, 0.9)). In this case, starting from two very close initial conditions (either (*M*, *A*, *R*)(0) = (2.1, 4.0, 5.0), (2.2, 4.0, 5.0) or (*M*, *A*, *R*)(0) = (2.1, 0.1, 0.1), (2.2, 0.1, 0.1)), the dynamics lead to either $T_{p}$ or $T_{m}$ phases. This dichotomous behaviors of the miR-451 (*M*), AMPK complex (*A*), and mTOR (*R*) modules in our mathematical model in response to high, intermediate, and low glucose levels are in good agreement with biological observations in [6, 18] (and other references therein), which leads us to the characterization of up- and down-regulation of these molecules relative to the threshold values (*th*_*M*_, *th*_*A*_, *th*_*R*_) for cell proliferation and migration in our modeling framework.

For simplicity, we assume that miR-451-AMPK-mTOR is the only signaling network that determines the cell proliferation and set *G*_0_ = 0 for the rest of sections. In the next section, we incorporate the core control system into the full hybrid model and analyze the full dynamics of glioma cell behavior in the presence of BVs.

### Dynamics of the hybrid model

**Figure 5:**
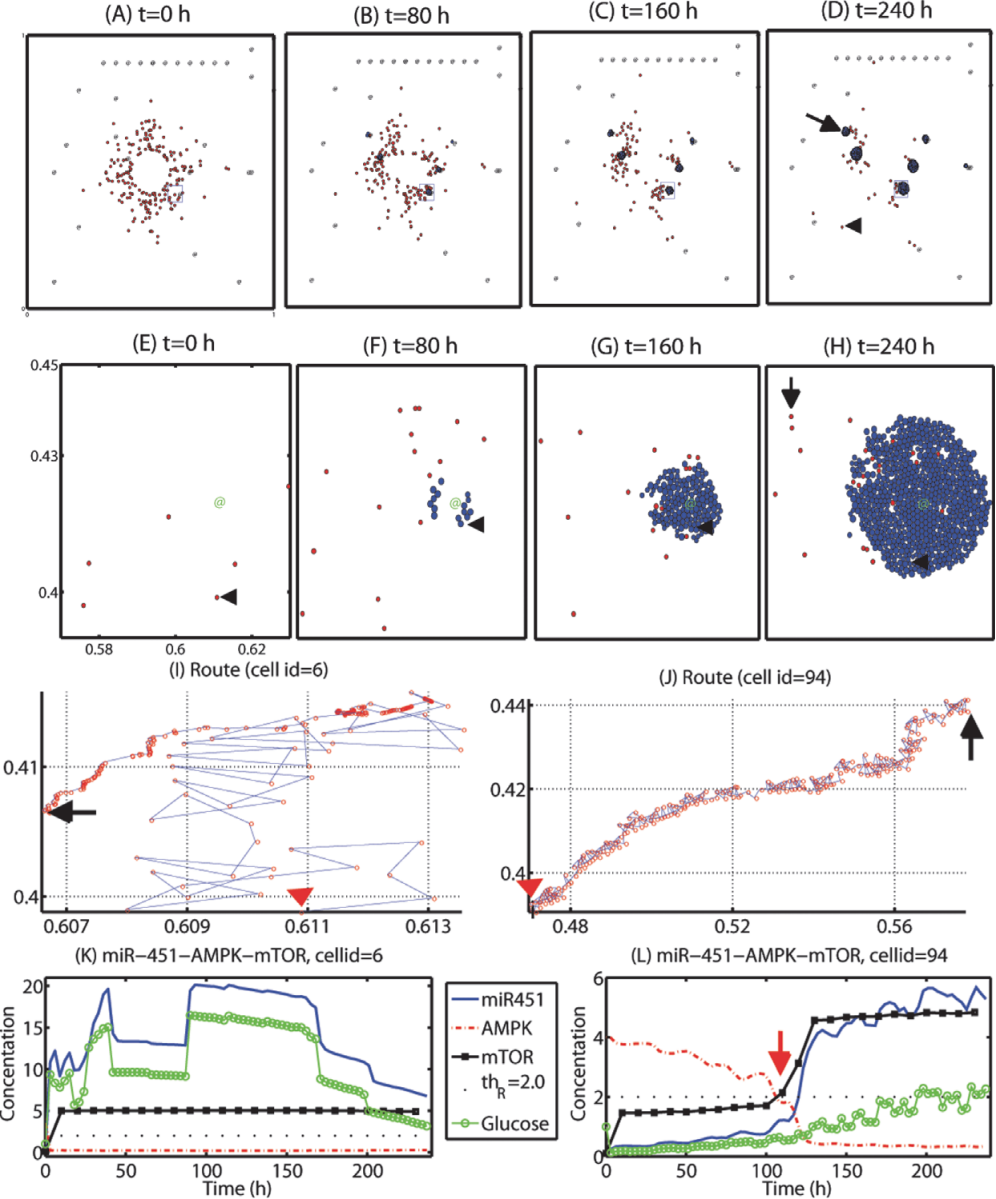
Dynamics of cell migration and proliferation in the hybrid model. Some diffusely infiltrating glioma cells in the $T_{m}$-phase are attracted to the glucose-rich BV sites and switch to the $T_{p}$-phase for fast growth in the favorable microenvironment while others are still moving around in the heterogeneous environment. (A-D) Tumor growth/invasion patterns in the computational domain Ω = [0, 1]^2^ at *t* = 0, 80, 160, 240 *h* after surgery. Arrowhead = migratory cell, arrow = growing cells near a BV. @ = BV. (E-H) Profiles of tumor cells in a small region [0.57, 0.63] × [0.39, 0.45] (blue solid box in (A-D)) of the domain. Arrowhead = 6th cell, arrow = 94th cell. @ = BV. (I, J) Migration routes of two cells (cellid = 6 in (I), 94 in (J)). Red arrowhead = initial position, Black arrow = final position at *t* = 240 *h*. (K, L) Concentrations of miR-451 (blue solid line), AMPK complex (red dotted line), mTOR (black circle), and glucose level at the cell site of two cells considered in (I, J). Threshold value of mTOR (*Th*_*R*_ = 2.0) was represented in black dots. Legends in (L) is same as one in (K).

Figs. 5A–5D show tumor growth and associated invasion patterns in the computational domain Ω = [0, 1]^2^ at *t* = 0, 80, 160, 240 *h* after surgical resection of the main tumor mass at the center of the domain at *t* = 0 *h*. Invisible invasive cells (red circles) respond to the gradient of glucose (∇*G*) and randomly move with a probability of *ψ*_1_ = 0.6. Most of cells settle in one of BV sites (for instance, see black arrow in Fig. 5D) while some cells are still in the migratory phase (black arrowhead in Fig. 5D). See Figure S5 in Supplementary File for the corresponding spatial profiles of concentrations of diffusive molecules. Figs. 5E–5H show spatial profiles of those cells near one of the BVs in a smaller frame ([0.57, 0.63] × [0.39, 0.45]; blue solid box in Figs. 5A–5D) at the same time frame. Once settled in the BV (marked in green @) region, these cells respond to high glucose levels from the BV and become proliferative cells (blue circles). Fig. 5K shows time courses of concentrations of miR-451 (blue solid line), AMPK complex (red dotted line), mTOR (black square), and glucose (green circle) for a growing cell near the BV (cellid = 6; black arrowhead in Figs. 5E–5H). Initially, low miR-451 level is increased and maintained above the threshold value (*th*_*M*_ = 2.0) in response to fluctuating but high glucose concentrations at the cell site as the cell is getting close to the BV site. This leads to down-regulation of AMPK complex (*A* < *th*_*A*_ = 2.0) and up-regulation of mTOR (*R* > *th*_*R*_ = 2.0; *th*_*R*_ was marked in dotted line). Moreover, a highly adaptive response of AMPK complex and mTOR can be observed in response to fluctuating high miR-451 levels. This over-expression of mTOR leads to a regular cell cycle and proliferation. Our simulation results are in good agreement with up-regulation of miR-451 expression among aggregates of tumor cells near BVs in biological experiments [6, 18].

At the later time (*t* > 175*h*), the glucose level, and consequently miR-451 is decreased. There are two possible reasons for this: (i) the cell is mechanically pushed away from the BV site (source of glucose) through mechanical interactions with other cells in the local neighborhood as the number of growing tumor cells is increased; (ii) as the tumor cells grow, the consumption of glucose by tumor cells is increased. This imbalance between supply and consumption of glucose as the tumor grows near the BV and translocation of the cell from mechanical interactions with other cells as it grows cause fluctuating glucose levels at the cell site overall. Random motility of the cell (*ψ*_1_ = 0.6) before it enters the BV region also contributes to the irregular glucose levels. See Fig. 5I for trajectory of the cell which was initially positioned away from the BV (red arrowhead) and passively positioned near the outer boundary of the growing tumor mass near the BV at the final time (*t* = 240 *h*; black arrow). When free space is still available (Fig. 5F), motile cells and growing cells are still moving around the BV. However, as the growing tumor cell density is increased (Fig. 5F→Fig. 5G→Fig. 5H), active migration of cells inside the growing tumor mass is completely turned off due to physical constraints. Growth of these cells surrounded by neighboring cells in the core is also partially suppressed due to growth function (*cf* Figure S3 in Supplementary Information) [20–23]. On the other hand, invasive cells which were initially positioned far away from BVs, did not settle down at one of those BVs during the simulation. (For instance, a cell (cellid = 94) marked with black arrow in Fig. 5H.) Fig. 5J shows the relatively long route of the traveling glioma cell (cellid = 94) from initial position (red arrowhead) to (black arrow). Despite the random motion occurring most of time, the cell migrate toward the same BV site as in Figs. 5E–5H in a consistent fashion. Fig. 5L shows concentrations of variables in the core miR-451-AMPK-mTOR system. As the cell approaches the BV, the glucose level at the cell site is slowly increased and the biochemical signal in migratory phase (*M* < *th*_*M*_, *A* > *th*_*A*_, *R* < *th*_*R*_) switches to the signal in proliferative phase (*M* > *th*_*M*_, *A* < *th*_*A*_, *R* > *th*_*R*_) around *t* = 110 *h*. However, this cell is still in the migratory mode at the final time (*t* = 240 *h*) since it did not reach the BV site yet. Therefore, a biomechanical switch for cell fate, i.e., proliferation and migration, may play a significant role in developing an anti-cancer drug or determining tumor-healthy tissue margins for any followup surgical resection of the recurrent tumors.

In the next section, we explore the glioma growth and invasion patterns for various random cell motility parameters (*ψ*_1_) and different BV densities, and investigate the effect of conventional chemotherapy drugs on killing migratory cells under these conditions.

### Glioma cell invasion and efficacy of chemotherapeutic after surgery

**Figure 6:**
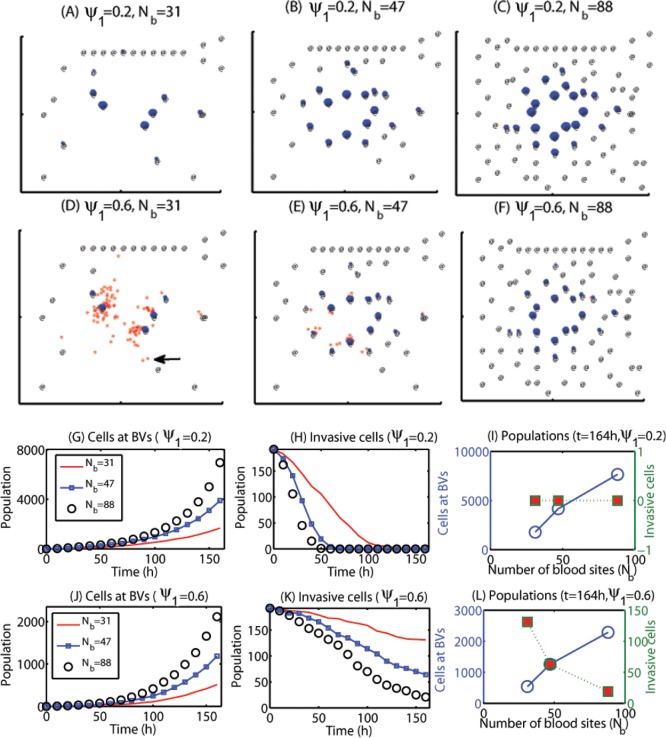
Effect of random motility and blood density on regulation of migration patterns. The tumor population is increased regardless of the relative ratio of the random motility (*ψ*_1_) as the number of blood vessels (*N*_*b*_) is increased because more invasive cells settle down near BVs, leading to faster tumor growth. On the other hand, fewer infiltrative cells switch to the growth phase for the relatively low BV density in the case of greater cell motility, reducing overall tumor growth but increasing further infiltration potential. (A-C) Spatial patterns of migratory cells (red dots) and growing cells near BVs (blue dots) at *t* = 164 *h* for a low (*ψ*_1_ = 0.2) random motility in the presence of various densities of blood vessels (*N*_*b*_ = 31, 47, 88) in the brain tissue after surgery. @ = BV sites. (D-F) Same as (A-C) but for an intermediate level of random motility (*ψ*_1_ = 0.6). (G-I) Time courses of populations of growing cells at BVs (G) and invasive cells distant from BVs (H) for three cases in (A-C) with the low (*ψ*_1_ = 0.2) random motility: (*N*_*b*_ = 31 (red solid line), 47 (blue square), 88 (black circle)). Populations of proliferative (blue empty circles) and invasive (red filled squares) tumor cells at final time *t* = 164 *h* for various *N*_*b*_ (*N*_*b*_ = 31, 47, 88) are shown in (I). (J-L) Same as (G-I) but for an intermediate level of random motility (*ψ*_1_ = 0.6). The legend is same as one in (G-I).

Figs. 6A–6C show spatial profiles of migratory cells (red dots) and growing cells near BVs (blue dots) at *t* = 164 *h* for various BV density when the random motility parameter was set *ψ*_1_ = 0.2. Here the number of BV sites was increased (from *N*_*b*_ = 31 in (A), to *N*_*b*_ = 47 in (B), and to *N*_*b*_ = 88 in (C)) with relatively uniform densities in the computational domain. Figs. 6G–6H show time courses of populations of growing cells near BVs (in (G)) and invasive cells away from BVs (in (H)) for these three cases (*N*_*b*_ = 31 (red solid line), 47 (blue square), 88 (black circle)). As the BV density is increased, population of growing cells near BVs is increased and the number of invasive cells away from BV sites is decreased since the chance of being attracted to BVs is increased. At the final time (*t* = 164 *h*), there are no invasive cells left for these three cases (red squares in Fig. 6I) but the number of cells at BVs is increased as *N*_*b*_ is increased. When the random motility parameter (*ψ*_1_) is set to be an intermediate value (*ψ*_1_ = 0.6), the overall dynamics of changes in populations of migratory cells and proliferative cells near BVs is similar to the case in *ψ*_1_ = 0.2. See Figs. 6J–6K. However, the chance of settling in BVs is decreased due to the increased random motility (from *ψ*_1_ = 0.2 to *ψ*_1_ = 0.6). For example, a migratory cell (black arrow in Fig. 6D) did not settle in one of BVs when *ψ*_1_ = 0.6 and blood density is relatively low (*N*_*b*_ = 31). However, these migratory cells eventually settle in one of BVs when blood density is high (*N*_*b*_ = 88 in Fig. 6F).

**Figure 7:**
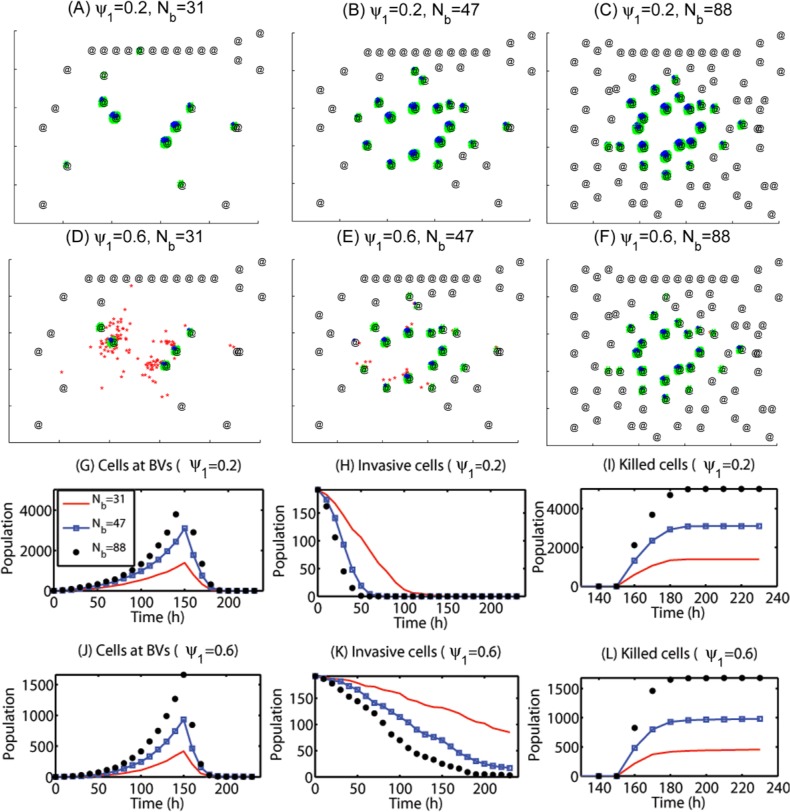
Effect of chemo-drug on killing migratory cells. Chemotherapy is highly effective for growing cells near BVs but the treatment efficacy is low for highly infiltrative glioma cells (high *ψ*_1_) due to low permeability at BVs (BBB). (A-C) Spatial patterns of glioma cells at *t* = 164 *h* for a low (*ψ*_1_ = 0.2) random motility in the presence of various densities of blood vessels (*N*_*b*_ = 31, 47, 88) after intravenous injection of S-phase targeting-Chemo-drugs at *t* = 150*h*. Red = migratory cells, blue = growing cells near BV sites, green = apoptotic cells due to the chemo-drug. @ = BV sites. (D-F) Same as (A-C) but for an intermediate level of random motility (*ψ*_1_ = 0.6). (G-I) Time courses of populations of growing cells at BVs (G), invasive cells away from BVs (H), and killed tumor cells near BV sites (I) for three cases in (A-C) with the low (*ψ*_1_ = 0.2) random motility: (*N*_*b*_ = 31 (red solid line), 47 (blue square), 88 (black circle)). (J-L) Same as (G-I) but for an intermediate level of random motility (*ψ*_1_ = 0.6). The legends in (H-L) are same as one in (G).

In Fig. 7 we illustrate the effect of chemotherapy on killing cancerous cells for all cases in Fig. 6. A S-phase targeting chemo-drug was given intravenously at *t* = 150 *h*. Figs. 7A–7C and 7D–7F show spatial profiles of cancerous cells in the case of different BV densities at *t* = 164 *h* for low (*ψ*_1_ = 0.2) and intermediate (*ψ*_1_ = 0.6) random motilities, respectively. Figs. 7G–7I show time courses of populations of growing tumor cells near BVs (Fig. 7G), invasive tumor cells away from BVs (Fig. 7H), and apoptotic tumor cells responding to the chemo-drug (Fig. 7I). Figs. 7J–7L show the time courses of populations when the random motility is strengthened (*ψ*_1_ = 0.6). Despite different numbers of tumor cells and subgroups near BVs, significant number of cancer cells near BVs for all six cases are killed already for all six cases due to relatively easy access of the drugs near BVs (green dots in Figs. 7A–7F; Figs. 7G, 7J). However, the killing rates of invasive cells (red dots Figs. 7A–7F) away from BV depend on the random motility (*ψ*_1_) and BV densities. When the random motility is low (*ψ*_1_ = 0.2), all migratory cells become growing cells at the BV sites and therefore respond to chemotherapeutic regardless of BV densities (Figs. 7A–7C). On the other hand, when the random motility is increased (*ψ*_1_ = 0.6) and BV density is low (*N*_*b*_ = 31), some of the migratory cells do not settle in a BV site and are not killed due to low accessibilities of chemotherapeutic far from BV sites (red dots in Fig. 7D; red solid curve in Fig. 7K). As the BV density is slightly increased (*N*_*b*_ = 47), the number of drug-free invasive cells is decreased but these survived cells are sources of potential recurrence even after chemotherapy, decreasing overall longterm survival rate of the glioma patients. However, the BV density is further increased (*N*_*b*_ = 88), all tumor cells respond to the chemo-drug and are killed since all migratory cells are localized near BVs. These results imply that depending on the changes in microenvironment near the origin of the main glioma mass, different appropriate strategies of chemotherapy should be applied to glioma patients. The blood brain barrier (BBB), a separation of circulating blood from the brain extracellular fluid (BECF) in the central nervous system (CNS), also plays a significant role in regulation of diffusion of the chemo-drugs at BV sites, i.e., transport of the drugs to brain tissue. Therefore, the BBB in addition to spatial distribution of BVs near the tumor site would affect the efficacy of chemotherapeutic after surgical resection of the main glioma core.

Tumor recurrence after conventional surgery is a major obstacle in treating glioblastoma. In the next section, we propose various localization strategies as a way of eradicating the *invisible* migratory cancer cells in combination with chemotherapy after surgery.

### Predictions of the model for a possible therapeutic approach

In this section, we developed therapeutic strategies for eradicating *invisible* migratory glioma cells in the brain tissue after conventional surgery where only *visible* parts of the tumor mass are removed. Here we assume that invasive cells in the surrounding tissue can sense and respond to the chemoattractant gradient (∇*C*) as well as glucose gradients created from BV sites.

**Figure 8:**
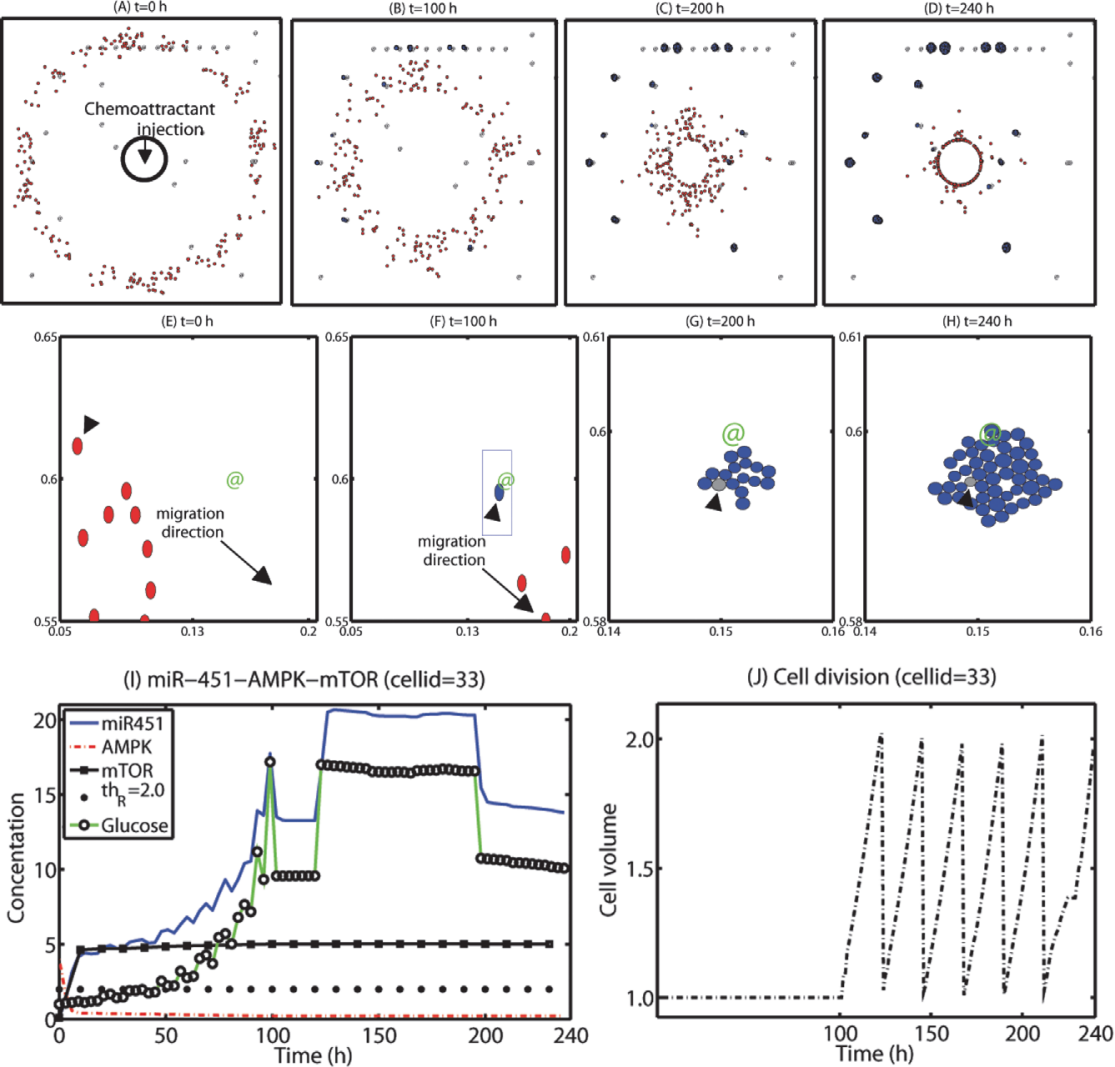
A proposed *localization* strategy of eradicating *invisible* migratory cells after first surgery. Some infiltrative glioma cells move back to the periphery of the resection site for the second surgery in response to the injected chemoattractant at the resection site after conventional surgery. However, some growing cells near BVs remain near BVs and do not respond to the chemotactic forces, reducing the efficacy of the localization strategy. (A-D) Spatial profile of cancerous cells at *t* = 0 (A), 100 (B), 200 (C), 240 (D) *h* after surgical resection at *t* = 0*h* followed by an injection of a chemoattractant at the resection site. The visibly detectable tumor core was surgically removed at *t* = 0 *h* (black solid circle in (A)). The localized invasive (red circles) glioma cells form a mass of tumor in (D) for a possible secondary surgery after 10 days, leading to eradication of invisible migratory cells. However, some of the cells (blue circles) attracted to BVs can still grow. Domain size = [0, 1]^2^. (E-H) Spatial profiles of a subset of migratory cells. Red circles = migratory cells, blue circles = growing cells, @ = BV. (I-J) A time course of miR-451-AMPK-mTOR (I) and cell volume in a growing glioma cell (arrowheads in (E-H); cellid = 33) near a BV in response to high glucose from the BV.

**Figure 9:**
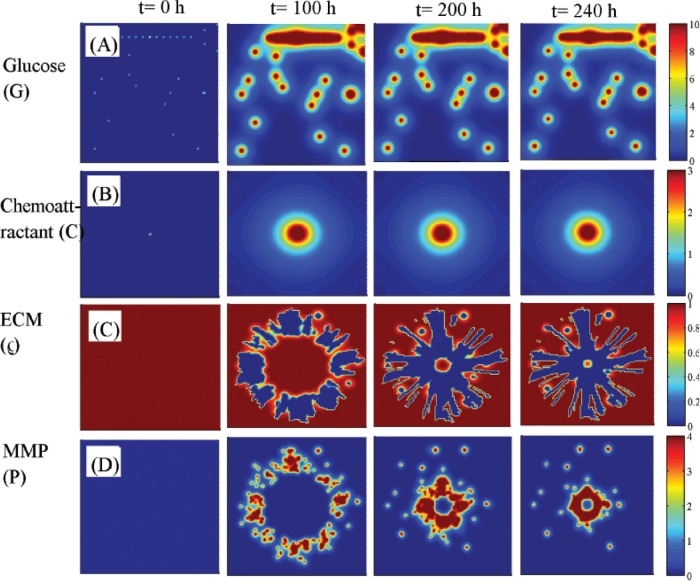
Dynamics of diffusible molecules. (A-D) Spatial profiles of glucose (*G* in (A)), chemoattractant (*C* in (B)), ECM (*ρ* in (C)), and MMP (*P* in (D)) at *t* = 0, 100, 200, 240 *h* that corresponds to Figs. 8A–8D. Glucose fluxes are provided at BV sites. The chemoattractant was injected at the center of the domain (0.5, 0.5). Domain size = [0, 1]^2^. Glucose is continuously supplied through BVs while chemoattractants diffuse through the domain after strategic injections at the center to attract infiltrative glioma cells. ECM is degraded and undergoes constant remodeling via MMPs secreted by migratory tumor cells toward the center during the localization process.

We first test the efficacy of the localization strategy without chemotherapy on killing invasive glioma cells. Figs. 8A–8D show spatial profiles of the invasive tumor cells at *t* = 0, 100, 200, 240 *h*, respectively. After first surgery (region inside black solid circle in Fig. 8A) at *t* = 0, a chemoattractant was injected at the center (0.5, 0.5) of the resected area. See Fig. 9B for spatial profiles of chemoattractant at the corresponding time frames *t* = 0, 100, 200, 240 *h*. Even though migratory cells (red circles) do the random walk (*ψ*_1_ = 0.2), they begin to migrate back to the tissue near the resection site. On the other hand, high glucose levels trigger the intracellular switch from the migratory phase to the proliferative mode for invasive cells near the BV site (Fig. 9A). BV sites were marked in @. Figs. 8E–8H show close-up profiles of movement of glioma cells in smaller subframes ([0.05, 0.2] × [0.55, 0.65] in (E-F); [0.14, 0.16] × [0.58, 0.61] in (G-H)) in Figs. 8A–8D. while most of other invasive cells (red circles) are migrating toward (black arrows) the resection site due to injection of a chemoattractant, a glioma cell (cellid = 33; black arrowheads) settles in a BV site on the routes of the migratory path (Fig. 8F). The cell near a BV (green @) then settles in a comfortable environment with enough glucose and begins to grow again. Figs. 8G–8H show growth of cancerous cells near the BV in a zoom-up subframe (blue box in Fig. 8F). Blue cells in Figs. 8G–8H show progenies of the first immigrant cell to arrive at the BV (gray cell; black arrowheads).

Figs. 8I–8J show time courses of the core control module (miR-451, AMPK, and mTOR) and cell volume at a cell site (cell id = 33; arrowheads in Figs. 8E–8H) in response to fluctuating glucose levels due to its proximity to BV on the way back to the resection site. At the beginning of the simulation (*t* = 0*h*), when a glioma cell is randomly searching for a better environment, both miR-451 and mTOR levels are low and the AMPK level is high due to the relatively low glucose level in the environment. As the cell approaches the BV site, the high glucose level (green line with black marker) induces over-expression of miR-451 levels (blue solid line) and decrease in AMPK activities (red dotted line) leading to up-regulation of mTOR levels (black square). The threshold value of mTOR (*th*_*R*_ = 2.0) was set in a black dotted line. The glucose level is increased during its migratory phase toward the BV site and maintains a relatively plateau state after stalling the migration and initial cell divisions but slightly decreased after *t* = 200 *h* due to translocation from the mechanical interactions with the neighboring daughter cells and increased consumption of glucose by the growing mass of these tumor cells. This switch to the growth phase leads to downstream signals to generate the regular cell cycle for this growing cell (Fig. 8J). As the number of daughter cells increases the original cell (black arrowhead) is surrounded by other cells and the duration of the cell cycle is slightly increased due to partial growth arrest from mechanical constraints after *t* > 200 *h*. Over-expression of miR-451 and proliferation of glioma cells near BVs were observed [6]. While infiltrative tumor cells after surgery may not be detected visually, a localized mass of invasive tumor cells may be detected by conventional screening tools such as MRI and could be surgically removed or eliminated by radiotherapy. This would increase the probability of eliminating invasive cells. However, localization of these cells to the resection site may not be enough and growing cells near BV sites may have to be removed as well in this case. Fig. 9 shows spatial profiles of glucose (*G* in Fig. 9A), chemoattractant (*C* in Fig. 9B), ECM (*ρ* in Fig. 9C), and MMP (*P* in Fig. 9D) at *t* = 0, 100, 200, 240 *h* that corresponds to Figs. 8A–8D. Glucose fluxes are provided at BV sites. The chemoattractant was injected at the center (0.5, 0.5) of the domain [0, 1]^2^.

**Figure 10:**
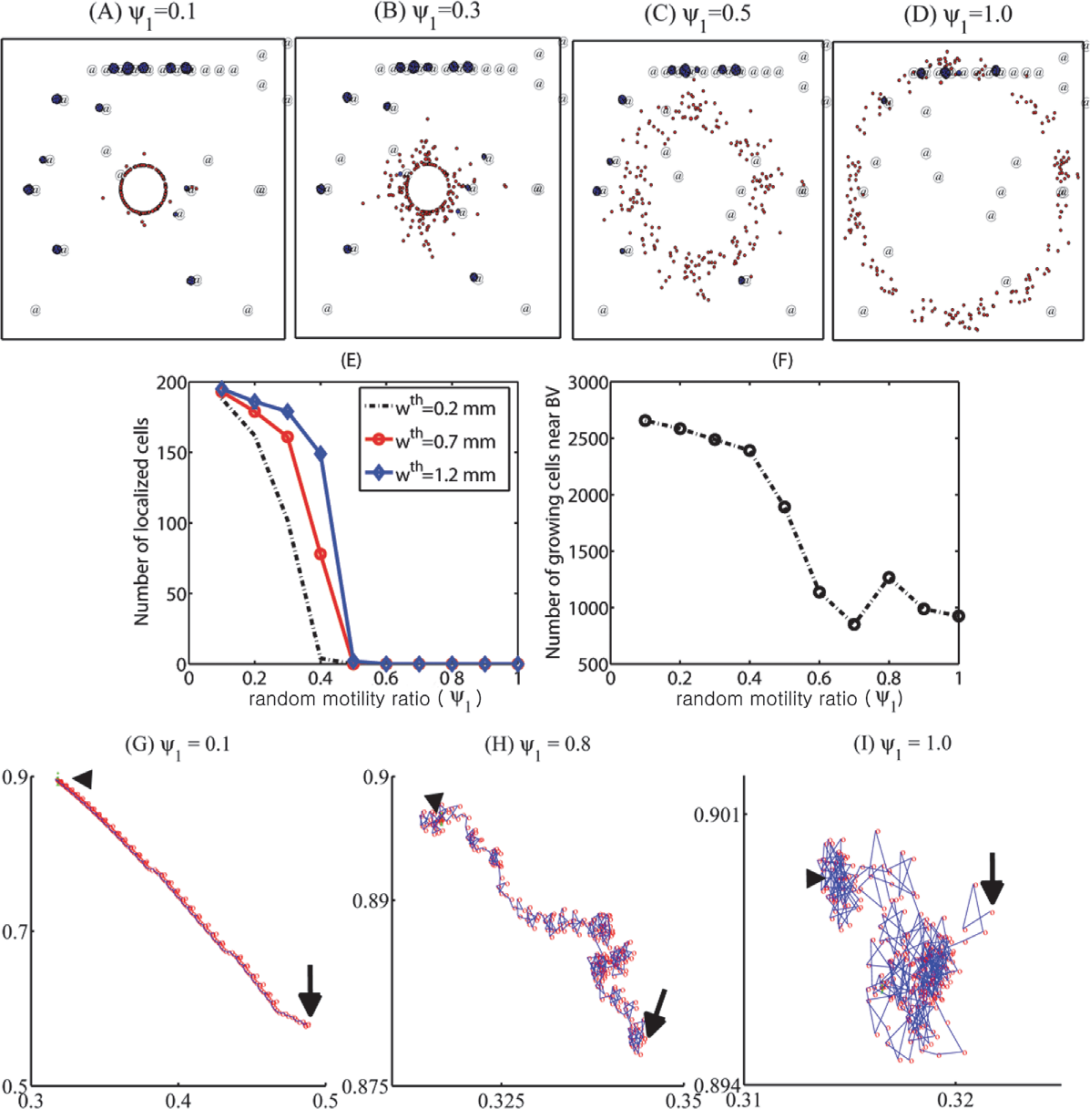
Effect of random motion on the efficacy of localization strategies. (A-D) Patterns of cell migration under the localization strategy with various random motility rates: *ψ*_1_ = 0.1 (A), 0.3 (B), 0.5 (C), 1.0 (D). (E) Population of localized invasive cells at day 10 within a narrow strip (Ω_*p*_) on the periphery of the resected area (Ω_*s*_) from the first surgery in the whole domain (Ω). Ω_*p*_ = {*P* = (*x*, *y*) : *dist*(*P*, Ω_*s*_) < *w* ^*th*^} where *w* ^*th*^ = 0.2, 0.7, 1.2 *mm*. (F) Population of proliferative cells at BV sites after chemoattractant injection as in (E) at final time (*t* = 10 days). (G-I) Trajectories of migratory cells in the brain tissue when *ψ*_1_ = 0.1 (G), 0.8 (H), 1.0 (I). Arrowhead = initial position, arrow = end position. As random motility *ψ*_1_ is increased, the relative chemotaxis strength is decreased and infiltrative glioma cells do not respond to the chemoattractant signals, reducing the treatment efficacy of localization strategies.

Random motility of glioma cells affect treatment strategies since unpredictabilities of migration direction might affect localization. Figs. 10A–10D shows different patterns of glioma cell localization with various random motility of glioma cell migration (*ψ*_1_ = 0.1 (Fig. 10A), 0.3 (Fig. 10B), 0.5 (Fig. 10C), 1.0 (Fig. 10D). As the motility parameter (*ψ*_1_) is increased, the efficacy of localization of invasive cells back to the resection site is decreased (Fig. 10E) but the population of growing cells near BV sites also is decreased. Figs. 10G–I show trajectories of an invasive cell (cell id = 1) with a starting point (0.32, 0.89) when *ψ*_1_ = 0.1 (10G), 0.8 (10H), 1.0 (10I).

**Figure 11:**
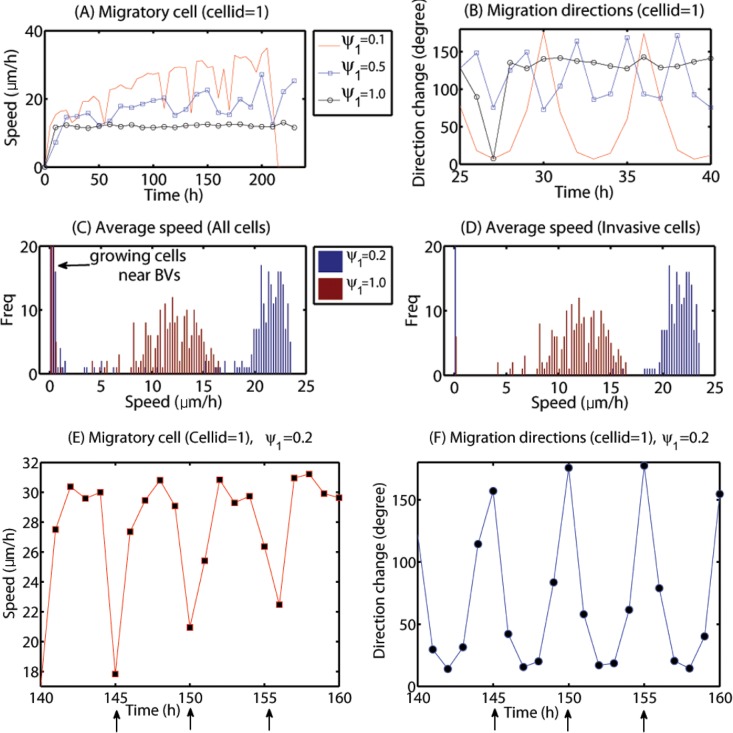
Effect of the random motion on cell speeds and directions. (A, B) Cell speed and moving direction of a migratory cell (cell id = 1; Figs. 10G–10I) for small (*ψ*_1_ = 0.1; red solid line), intermediate (*ψ*_1_ = 0.5; blue square) and large (*ψ*_1_ = 1.0; black circle) random motility. (C, D) Distributions of average speeds of all cells (C) and invasive cells (D) for small (*ψ*_1_ = 0.2; blue) and large (*ψ*_1_ = 1.0; red) random motility. (E, F) Cell speed and direction changes of the cell (cell id = 1) over time interval [140, 160] in hours for the base random motility (*ψ*_1_ = 0.2). The black arrows indicate the location and corresponding times at which the cell changed direction. Fluctuating speeds within a biologically observed range [27, 29–31] and saltatory patterns in the migration direction are consistent with experimental data in a study by Farin *et al*. [27].

To test the validity of the model, we measure cell speeds and patterns of migration direction changes for various random cell motility parameters (*ψ*_1_) in order to compare them with experimental data. Fig. 11A shows time courses of the cell speed and direction change (degree) of a migratory cell responding to the chemotactic signal near the resection site (cellid = 1; Figs. 10G–I) with various random motility levels: *ϕ*_1_ = 0.1 (red solid line), *ϕ*_1_ = 0.5 (blue square) and *ψ*_1_ = 1.0 (black circle). When the random motility is small (*ψ*_1_ = 0.1), the cell speed fluctuates between values in the range of (18–36) *μm*/*h* until the cell speed begins to drop down to zero around∼212 *h* due to cell aggregation near the resection site. For the intermediate case (*ψ*_1_ = 0.5), the cell speed still fluctuates in the lower range (16–32 *μm*/*h*) but the cell did not arrive at the resection site and still maintains the high speed at the final time (*t* = 10 *days*). When the random motility ratio is 1, the cell does not respond to the chemoattractant signal at all and persists the random walk motion with the relatively lower cell speed (8–14 *μm*/*h*). Fig. 11B shows direction changes (degree) of the migratory cells with various random motilities (*ψ*_1_ = 0.1, 0.5, 1.0) in Fig. 11A. Direction changes become more frequent as the random motility parameter (*ϕ*_1_) is increased. Figs. 11E–11F show cell speeds and direction changes in degrees of the same cell (cellid = 1) over a time interval [140, 160] (in hours) for the base random motility in the model (*ψ*_1_ = 0.2). The cell speed fluctuates between low value (18 *μm*/*h*) and high value (31 *μm*/*h*) and has the low value when the direction change (degrees) is high (black arrows in Figs. 11E–11F). These fluctuating speeds of migratory glioma cells and patterns of migration direction changes are consistent with experimental data in a study by Farin *et al*. [27] where cell speeds and trajectories of glioma cells were measured after injection of the eGFP and DsRed-2 labeled cells into neonatal rat forebrains. In this study the highest cell speed was 100 *μm*/*h* and the average speed of all cells was measured to be 24.7±0.8 *μm*/*h*. Figs. 11C–11D show distributions of the average speed of all cells (Fig. 11C) and migratory cells (Fig. 11D) for small (*ψ*_1_ = 0.2; blue) and large (*ψ*_1_ = 1.0; red) random motility. High localization of very low speeds in Fig. 11C accounts for proliferative cells near BVs, the progenies of small number of invasive cells that settled in BV sites. The low cell speed is because of the movement due to growth, not cell movement. While large random motility induces relatively high speeds (18–23 *μm*/*h*), small random motility leads to relatively lower cell speeds. A wide range of cell speeds of glioma cells in various biochemical and biomechanical conditions have been reported: 39–45 *μm*/*h* (2D barrier-free culture condition) and 15–20 *μm*/*h* (3D culture in the absence/presence of EGF-stimulation) [29], 15–25 *μm*/*h* (with/without *α*-actinin isoforms) [30], 5–80 *μm*/*h* (neonatal rat forebrain) [27], 15–48 *μm*/*h* (in collagen I matrix) [31]. Therefore, the cell speeds in our model simulations are in good agreement with experimental data.

**Figure 12:**
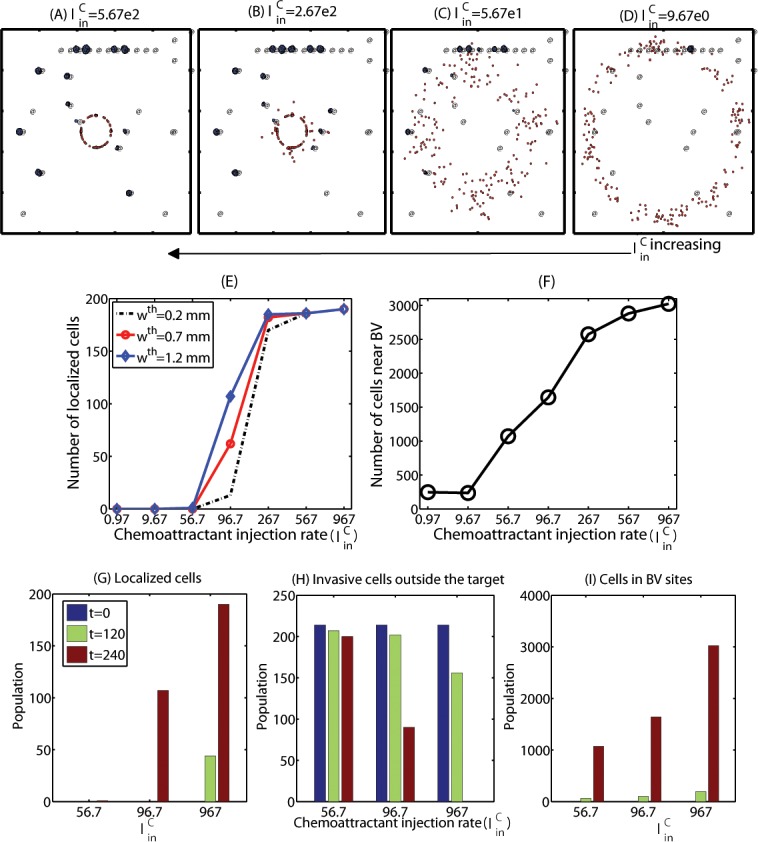
Localization efficacy in response to various chemoattractant injection rates. (A-D) Localization patterns of cancer cells at the final time point (*t* = 10 days) in response to injections of chemoattractants on the periphery of the resection site with the various injection rates ($l_{in}^{C}=5.67\text{e}2$ (A), 2.67e2 (B), 5.67e1 (C), 9.67 (D)). *N*_*C*_ = 1. (E) Population of localized invasive cells at day 10 within a narrow strip (Ω_*p*_) on the periphery of the resected area (Ω_*s*_) from the first surgery in the whole domain (Ω). Ω_*p*_ = {*P* = (*x*, *y*) : *dist*(*P*, Ω_*s*_) < *w* ^*th*^} where *w* ^*th*^ = 0.2, 0.7, 1.2 *mm*. (F) Population of proliferative cells at BV sites after chemoattractant injection as in (E) at final time (*t* = 10 days). Parameters: *ψ*_1_ = 0.2, *ψ*_2_ = 0.0, *ψ*_3_ = 1.0. (G-I) Populations of localized cells (G), invasive cells outside the target (H), proliferative cells in BV sites (I) at *t* = 0, 120, 240 *h*. While the localization efficacy is increased in part as chemoattractant injection rate ($l_{in}^{C}$) is increased, there are still growing cells near BVs without additional treatment.

**Figure 13:**
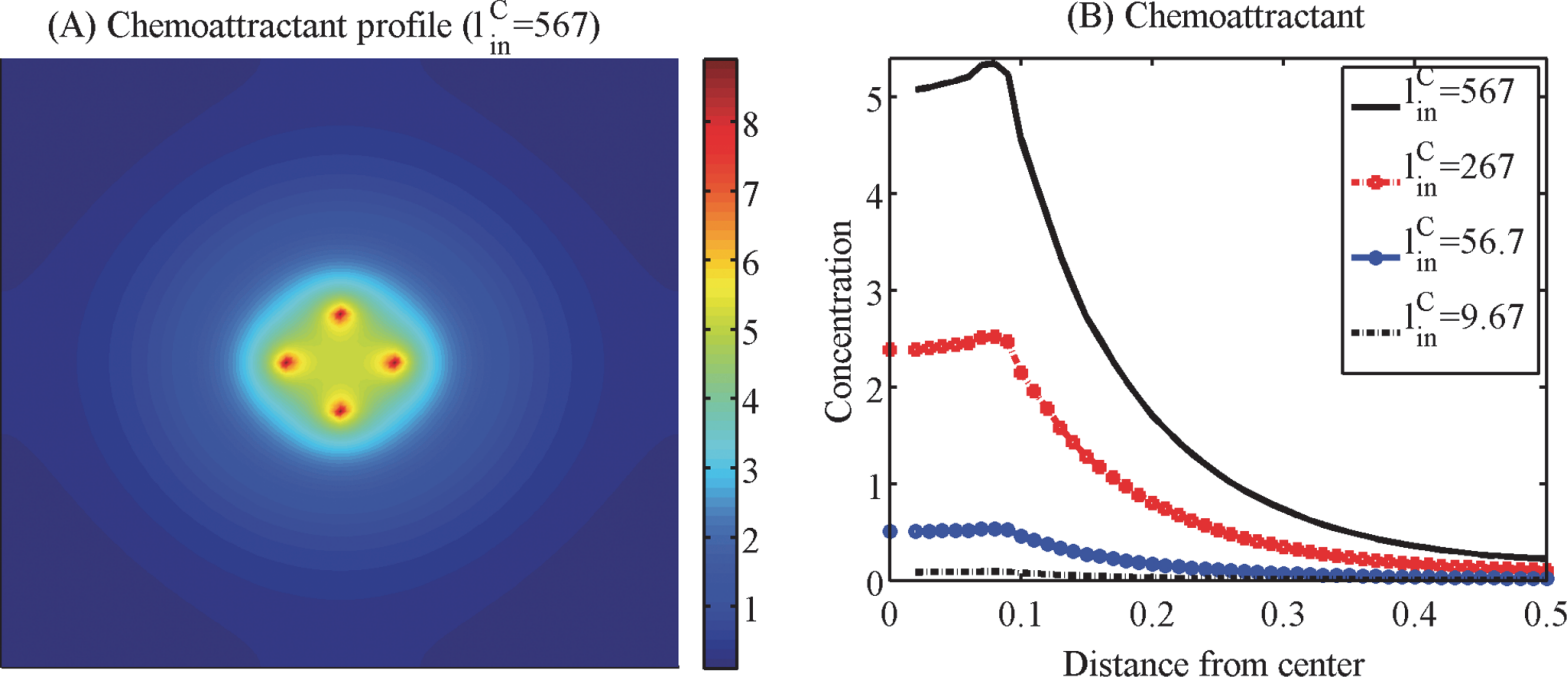
Spatial profiles of the chemoattractants near the surgical site. (A) Spatial profile of the chemoattractant at a high injection rate ($l_{in}^{C}=567$) in Fig. 12A. (B) Concentrations of the chemoattractant in the radial direction for four different injection rates ($l_{in}^{C}=567$, 267, 56.7, 9.67) in Figs. 12A–D. Relatively strong chemoattractant injections are necessary in order to create a sufficient gradient of chemoattractants to attract infiltrative glioma cells.

Efficacy of the localization method would also heavily depend on practical injection strategies of chemoattractants and therefore we next investigated the optimization of injection strategies. Figs. 12A–12D show localization patterns of cancer cells 10 days after injections of chemoattractants with the various injection rates ($l_{in}^{C}=5.67\text{e}2$ (A), 2.67e2 (B), 5.67e1 (C), 9.67 (D); *N*_*C*_ = 1). A chemoattractant was injected at four sites ((0.58, 0.5), (0.5, 0.58), (0.42, 0.5), (0.5, 0.42)) as point sources on the periphery of the resection site after conventional surgery. We notice that when large amount of a chemoattractant was introduced (as in Figs. 12A–B, 13A), dispersed invasive cells in the brain tissue bio-mechanically react to the injection of chemoattractants and migrate back to the injection sites on the periphery of the resection site at the center of the domain. However, invasive cells do not respond to the intervention in the case of low injection rates ($l_{in}^{C}=9.67$) as in Fig. 12D. Fig. 12E shows the population of localized invasive cells at day 10 within a narrow strip (Ω_*p*_) on the periphery of the resected area (Ω_*s*_) from the first surgery in the whole domain (Ω). Here, Ω_*p*_ = {*P* = (*x*, *y*) : *dist*(*P*, Ω_*s*_) < *w* ^*th*^} where *w* ^*th*^ = 0.2, 0.7, 1.2 *mm*. As the injection rate of chemoattractants ($l_{in}^{C}$) is increased, the number of localized cells is increased. For instance, most invasive tumor cells except cells trapped in BV sites migrated back to the resection site in response to the injection at a high rate ($l_{in}^{C}=567$; Fig. 12A). None of the invasive cells respond to the *weak* chemotactic signal in the case of the low injection rate ($l_{in}^{C}=567$; Fig. 12D). Figs. 12G–12I show population dynamics of localized cells (12G), invasive cells outside the target (12H), proliferative cells in BV sites (12I) at *t* = 0, 120, 240 *h*. We notice that an increase in $l_{in}^{C}$ leads to an increase in localized cells near the target area (resection site for the second surgery) and a decrease in the number of invasive cells in the brain tissue (thus potentially aggressive). However, the localization strategy via the chemoattractant injection does not prevent aggressive proliferation of glioma cells at several BV sites in the neighborhood. Therefore, an increase in injection rates of chemoattractants near the resection site leads to an increase in the number of aggressively growing cells at BV sites as well as shown in Fig. 12F. This indicates that a simple injection of chemoattractants near the resection site may not be sufficient and further additional strategies is necessary in order to prevent this unwanted rapid growth of tumor cells near BV sites. Fig. 13A shows a spatial profile of the chemoattractant when injected at a high rate ($l_{in}^{C}=567$). Fig. 13B shows concentrations of the chemoattractant in the radial direction for four injection rates ($l_{in}^{C}=567$, 267, 56.7, 9.67) corresponding to the cases in Figs. 12A–D.

**Figure 14:**
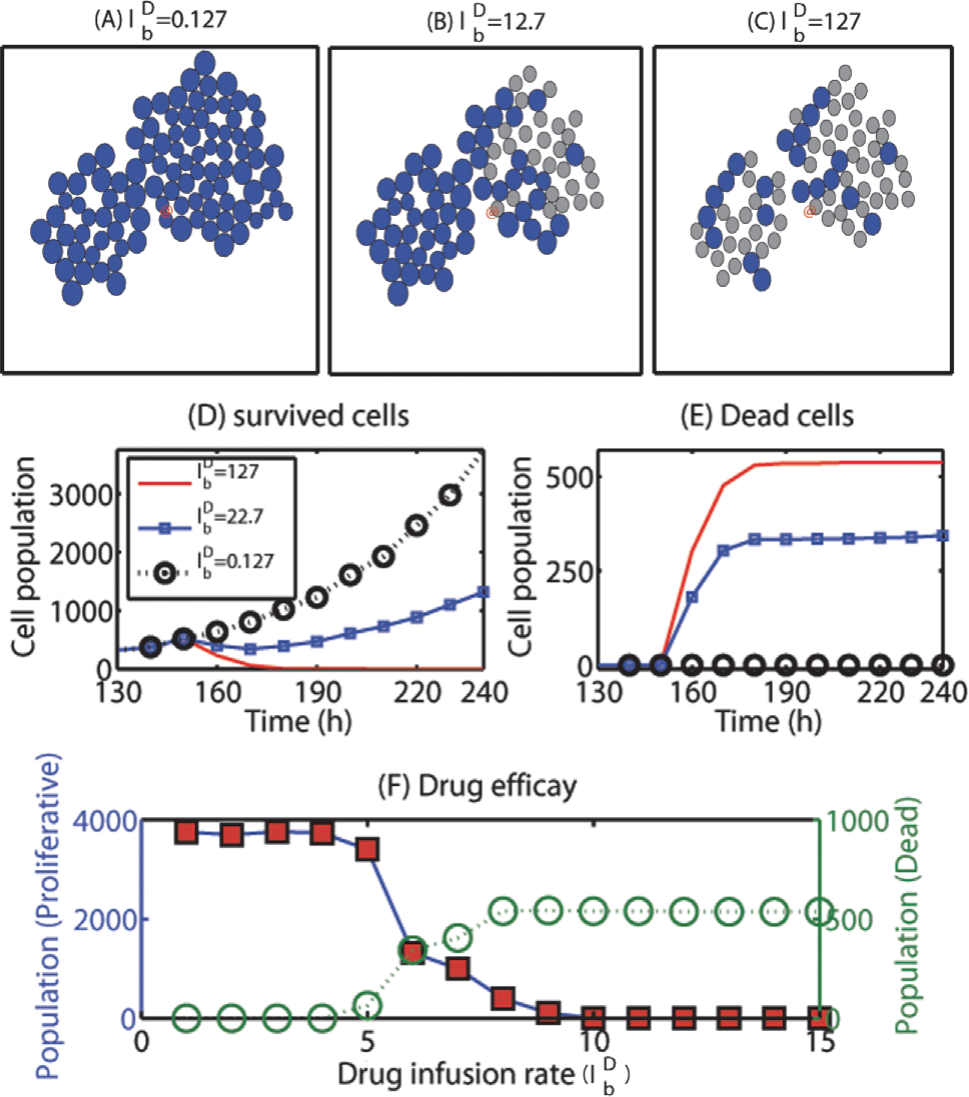
Effect of S-phase-targeting chemo-drugs on eradicating cancer cells. (A-C) Tumor migration-proliferation-apoptosis patterns at a BV site (@) at time *t* = 165 *h* in response to various intravenous infusion of a S-phase-targeting chemo-drug at *t* = 150 *h* in addition to localization of invasive tumor cells. While the intravenous infusion of the drug with a low infusion rate ($l_{b}^{D}=0.127$ in (A)) does not kill the cancer cells, larger amounts of drugs remove glioma cells in the S-phase. Blue ellipse = proliferative cells, gray ellipse = apoptotic cells. Frame size = [0.488, 0.512] × [0.89, 0.91] ⊂ [0, 1]^2^. (D, E) Time courses of populations of surviving actively growing cells (in (D)) and dead cells (in (E)) in response to three different dose rates ($l_{b}^{D}=0.127$ (dotted black circle), 22.7 (solid blue square), 127 (solid red line)). The legend in (E) is the same as one in (D). (F) Efficacy of killing cancerous cells by the S-phase-targeting drug: populations of proliferative (blue solid circle) and dead cells at the BV sites with various drug infusion rates ($l_{b}^{D}=1.27e-01(1)$, 5.27e-01(2), 1.27e+00(3), 5.27e+00(4), 1.27e+01(5), 2.27e+01(6), 3.27e+01(7), 4.27e+01(8), 5.27e+01(9), 6.27e+01(10), 7.27e+01(11), 1.27e+02(12), 5.27e+02(13), 1.27e+03(14), 3.27e+03(15)). As the drug infusion rate ($l_{b}^{D}$) is increased, the drug effectively kills the actively proliferative tumor cells at the BV sites. Parameters: *ψ*_1_ = 0.2, *ψ*_2_ = 0.0, *ψ*_3_ = 1.0. Intravenous infusion of a strong S-phase-targeting chemo-drug in addition to localization of cells may effectively kill growing cells near BVs, leading to complete eradication of tumor cells: infiltrative individual cells in the brain tissue and proliferative cells in a growing mass near BVs.

Now, we test our new hypothesis: intravenous administration of chemo-drugs in addition to localization of invasive cells would lead to better clinical outcomes in eradicating glioma cells. For this purpose, we introduce a chemo-drug that targets S-G2-M-phase in the cell cycle and diffuses in the brain tissue with sources at BVs after intravenous injection of the drug. Therefore, we introduce the following additional governing equation for the chemo-drug

$$\frac{\partial D}{\partial t}=\underset{\text{Diffusion}}{\underset{︸}{\nabla \cdot (D_{D}(x)\nabla D)}}+\underset{\text{Supply from blood}}{\underset{︸}{l_{b}^{D}I_{B}(x)}}-\underset{\text{Decay}}{\underset{︸}{\mu_{D}D}}$$ (22)

where *D*_*D*_ is the diffusion coefficient of the drug, $l_{b}^{D}$ is the intravenous infusion rate of the chemo-drug, and *μ*_*D*_ is the natural decay rate of the drug. In Fig. 14 we develop strategies to improve anticancer efficacy by intravenous infusion of a chemo-drug that targets *S*–*G*_2_–*M*-phase at *t* = 150 *h*. Figs. 14D, 14E show the population of survived and dead tumor cells, respectively, for various $l_{b}^{D}$ ($l_{b}^{D}=0.127$, 22.7, 127). With the low infusion rate ($l_{b}^{D}=0.127$; black empty circle), the efficacy is low and surviving cells near BVs will generate potential danger to the patients with regrowth or invade brain tissue as the re-formed tumor grows. For example, none of tumor cells near one of the BVs (marked in @) are responding to the chemo-drug at *t* = 165 *h* for the case of $l_{b}^{D}=0.127$ (Fig. 14A) while most of cells were dead with high infusion rate ($l_{b}^{D}=127$; gray cells in Fig. 14C; see red curve in Fig. 14E for dead cell population). As the drug infusion rate is increased, the anti-cancer efficacy is increased (Fig. 14F). Therefore, these results illustrate that the eradication strategy of invisible migratory cells by localization to the resection bed is not good enough and a combined strategy of intravenous infusion of chemo-drug for growing tumor cells near BVs and localization for migratory cells should be used in order to eradicate all the tumor cells.

**Figure 15:**
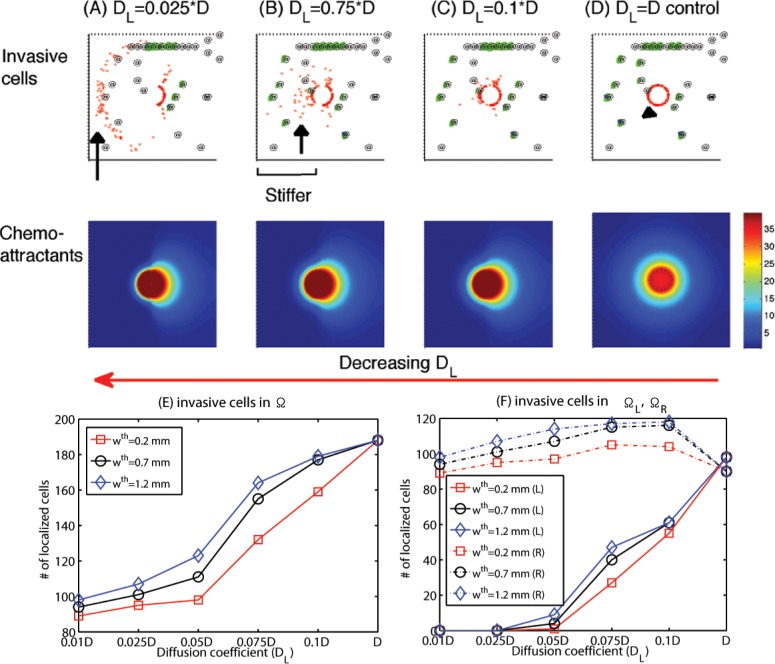
Role of the tumor cell microenvironment in efficacy of the treatment. (Top; A-D) Profiles of cancer cells at day 10 in response to injected chemoattractants on the periphery of the resection site in different tissue environment. (Bottom; A-D) Profiles of chemoattractants that correspond to each case in top panels. Diffusion coefficients of all variables were reduced on the left half side of the domain (Ω_*L*_ = [0, 0.5] × [0, 1]) while they were fixed on the right half domain (Ω_*L*_ = [0.5, 1] × [0, 1]) relative to control (*D*) in whole domain (in (D); Ω = [0, 1]^2^): *D*_*L*_ = 0.025*D* in (A), *D*_*L*_ = 0.075*D* in (B), *D*_*L*_ = 0.1*D* in (C), *D*_*L*_ = *D* in (D). As the diffusion coefficient *D*_*L*_ on the left half plane is decreased (from (D) to (A)), i.e., brain tissue is getting tougher, the efficacy of bringing those invasive cancerous cells back to the resection site is decreased. (E) Population of localized invasive cells within a narrow strip (Ω_*p*_) on the periphery of the resected area (Ω_*s*_) from the first surgery in the whole domain (Ω) at day 10. Ω_*p*_ = {*P* = (*x*, *y*) : *dist*(*P*, Ω_*s*_) < *w* ^*th*^} where *w* ^*th*^ = 0.2, 0.7, 1.2 *mm*. (F) Population of localized invasive cells within Ω_*p*_ in the left (Ω_*L*_) and right (Ω_*R*_) half domain at day 10. Parameters: *ψ*_1_ = 0.2, *ψ*_2_ = 0.0, *ψ*_3_ = 1.0. Efficacy of localization strategies depends on microenvironmental factors such as stiffness of tissue and brain tissue composition/geometry even with chemo-drugs since effective transport of key molecules (chemoattractants and chemo-drugs) by the diffusion process depends on these active microenvironmental players, requiring a careful priori assessment of patient-specific data.

Many biochemical factors can induce the stiffening of tissue; these include fibrosis and brain tumor growth [32], inhomogeneous endothelial transformation [33], and reorganization of ECM fibrils by differentiation and highly contractile stromal cells [34]. Hetergeneity in the tumor microenvironment leads to drug resistance [35], and reduced efficacy of tumor-targeting agents [36]. Therefore, it is important to observe the effect of microenvironmental factors on the localization strategies for patient-specific treatment options. In Fig. 15 we investigate the role of microenvironment in regulating the localization of the migratory glioma cells in response to chemoattractants injected near the resection bed at the center of the domain. In order to test the effect of tissue stiffness on the efficacy of the localization strategy, we prescribe lower diffusion coefficients (*D*_*L*_) on the left half domain (Ω_*L*_ = [0, 0.5] × [0, 1]) while keeping the normal (control) diffusion coefficients (*D*_*R*_ = *D*) in the right half domain (Ω_*L*_ = [0.5, 1] × [0, 1]): *D*_*L*_ = 0.025 * *D* (Fig. 15A), 0.075 * *D* (Fig. 15B), 0.1 * *D* (Fig. 15C), *D* (control; Fig. 15D). As *D*_*L*_ is decreased, some of migratory cells (black arrow in Figs. 15A, 15B) do not respond to the chemoattractant signals in the left half domain due to weak transport of the chemoattractant by diffusion (the lower panel in Figs. 15A, 15B). Figs. 15E, 15F show the population of invasive cells within a narrow strip in the whole domain (Ω) and subdomains (Ω_*L*_, Ω_*R*_), respectively, at day 10 for various diffusion coefficients in the left domain (*D*_*L*_ = 0.01 * *D*, 0.025 * *D*, 0.05 * *D*, 0.075 * *D*, 0.1 * *D*, *D*). Here the narrow strip (Ω_*p*_) is located on the periphery of the resected area (Ω_*s*_) from the first surgery in the whole domain (Ω) at day 10: Ω_*p*_ = {*P* = (*x*, *y*) : *dist*(*P*, Ω_*s*_) < *w* ^*th*^} where *w* ^*th*^ = 0.2, 0.7, 1.2 *mm*. As *D*_*L*_ is decreased, the population of localized tumor cells is significantly decreased in the left half domain (solid curves in Fig. 15F) while there is no significant difference in the right half domain (dotted curves in Fig. 15F), thus there is a significant decrease in the whole plane overall (Fig. 15E). This illustrates that the efficacy of the localization strategy also depends on the microenvironmental factor and the clinical outcomes will depend on tissue composition and location within the brain.

## Discussion

One of the major obstacles in treatment of GBM is that by the time the disease is diagnosed cancer cells have already spread into the neighboring brain tissue and the incomplete elimination of cancer cells by conventional therapeutic approaches leads to regrowth of these invasive cells, leading to the poor survival rate. Like the guerilla warriors, the glioma cells seem to posses specific characteristics that allow for diffusive infiltration [37]. Therefore, finding a way of blocking this critical invasion process or eradicating these *invisible* invasive tumor cells would lead to better clinical outcomes. In order to survive in the harsh microenvironment, glioma cells shift their metabolic machinery toward enhanced glucose uptake, *Warburg effect* [7–9], and cell migration to seek out better microenvironments (or through negative chemotaxis [38]) triggered by lowered glucose levels.

Multiple microsurgical resections for glioblastoma have been proven to be effective and useful [39]. However these do not prevent the tumor invasion before the surgery, posing a potential recurrence of the tumor. Using the hybrid model, we developed a localization strategy where tumor cells in the brain tissue are attracted back to the resection bed for the follow-up surgery or other treatments by injecting a chemoattractant on the periphery of the resection bed. Even though some of migratory cells are successfully localized to the resection bed, some of the migratory cells respond to the biochemical signals from BVs on the way back to the resection site and begin to grow in the neighborhood of the BVs via up-regulation of miR-451 and mTOR and down-regulation of the AMPK complex in response to high glucose levels from BVs (Fig. 5). Growth of the cells near a BV depends not only the signal status in the core control system but the physical constraints from the neighboring cells, *i.e.*, growth of cells in the interior of the growing tumor mass is inhibited [20, 21, 23].

Spatial patterns of localized cells near the resection bed and growing cells near BVs also depend on the BV density and the random motility (Fig. 6): lower density of BVs and lower random motility would result in the best outcomes. In the presence of a dense network of BVs, the effectiveness of localization strategy is decreased due to local attraction of cells to BVs while high random motility frees the cells from the chemotaxis intervention (Fig. 10). In general, intravenous infusion of chemo-drugs may not be effective due to BBB. However, in our framework, these *S*–*G*_2_–*M*-phase targeting chemo-drugs can effectively kill those tumor cells near BVs due to its relatively high accessibility to the tumor cells near BVs. Therefore, the model predicts that administration of the chemo-drugs in addition to the tumor cell localization may increase the efficacy of eradicating *all* invasive cells overall (Fig. 7). Our cell-based component within the hybrid model enable us to determine the speed, trajectories and directions of cell migration. The model predicts that the localization strategy also critically depends on the random motility of glioma cells (Fig. 11). The cell speeds of glioma cells were in good agreement with values in the measure values in the literature. More importantly, fluctuating cell speeds and migration directions of glioma cells are consistent with experimental observations under time-lapse microscopy in [27], which also observed localization of migratory cells at a BV followed by aggressive growth.

Invasive tumor cells can be cultured from biopsies up to 4*cm* away from the main bulk tumor [40]. When cancer cells migrate too far from the original resection site relative to the strength of the chemoattractants, it may be difficult to attract these migratory cells. For example, we found that too low dosage of the chemoattractant may not be able to attract migratory cells for the follow-up surgery (Fig. 12) and these *missed* cells would regrow later at the distant sites. Therefore, in order to attract those invasive cells in the far away field (> 4*cm* away) one might need to increase dosage of chemoattractants at the resection bed. In order to complement the drawbacks of the localization strategy, *i.e.*, regrowing cells that were trapped at a BV on the way back to the resection bed, we investigated the effectiveness of *S*–*G*_2_–*M*-phase targeting chemo-drugs on elimination of these regrowing cells. The model predicts that the whole strategy can eliminate both migratory cells near the resection bed and growing cells near BVs with enough dosage of chemo-drugs (Fig. 14).

The growth pattern of a GBM is formed based on perineuronal satellitosis, perivascular accumulation of cancer cells, intrafascicular growth in the corpus callosum, and subpial growth [37]. Therefore, the efficacy of anti-invasion strategies developed here also critically depends on where the primary tumor developed in the brain and accessibilities of drugs in the microenvironment it is in. Our model predicts that microenvironmental factors such as tissue stiffening are important to generate patient-specific feasible strategies for better clinical outcomes (Fig. 15). To avoid the unpleasant situation, one might have to inject chemoattractants at several sites in this case, requiring the follow-up surgery at several different positions. However, the strategy presented in this paper may serve as a novel way of eradicating all cancer cells when an appropriate combination of chemoattractants and chemo-drugs is used.

The analysis of the hybrid model in the current paper may serve as a starting point for further experimental investigation and more detailed modeling.

## Supporting Information

S1 File Strategies of eradicating glioma cells: A multi-scale mathematical model with miR-451-AMPK-mTOR control (PDF)
