# Supplementary material for: Strategies of Eradicating Glioma Cells: A Multi-Scale Mathematical Model with MiR-451-AMPK-mTOR Control
Source: PLoS One. 2015 Jan 28;10(1):e0114370. doi: 10.1371/journal.pone.0114370 (PMC4309536; doi:10.1371/journal.pone.0114370)
Supplement: S1 File — (PDF) [file pone.0114370.s001.pdf]

Supplementary Information File for "Strategies of eradicating glioma cells: A multi-scale mathematical model with miR-451-AMPK-mTOR control"

Yangjin Kim[1,2], Gibin Powathil[3], Hyunji Kang[1], Dumitru Trucu[3], Hyeongi Kim[4], Sean Lawler[5], Mark Chaplain[3,*]
1 Department of Mathematics, Konkuk University, Seoul, 143-701, Republic of Korea
2 Department of Mathematics, Ohio State University, Columbus, OH 43210, USA
3 Division of Mathematics, University of Dundee, Dundee, UK
4 Department of Physics, Konkuk University, Seoul, 143-701, Republic of Korea
5 Department of Neurosurgery, Brigham and Women's Hospital, Boston MA 02115, USA
*** E-mail: ahyouhappy@konkuk.ac.kr**

1 Derivation of the core control system (miR-451-AMPK-mTOR)

In this section, we develop a simple mathematical model for the regulation of the core miR-451-AMPK-mTOR system shown in Figure A. In order to incorporate the signaling network shown in Figure B(A) into our model, we simplified the network as shown in Figure B(B). By convention, the kinetic interpretation of arrows and hammerheads in the network represents induction (arrow) and inhibition (hammerhead). Previously, we developed a mathematical model of the miR-451-AMPK core control system based on the simplified network [18]. In this study, we extend our previous work to the miR-451-AMPK-mTOR system. We merged the entire regulatory network between CAB39/LKB1/STRAD and AMPK/MARK into one component (AMPK complex; blue dotted box in Figure B(A)) while we kept miR-451 in one module (brown dotted box in Figure B(A)). We also take into account the regulation of mTOR, which will play a significant role in linking miR-451-AMPK network to the cell cycle network. Let the variables m, a and r be activities of miR-451, AMPK complex, and mTOR, respectively. The scheme includes autocatalytic activities of miR-451 (m), AMPK complex (a), and mTOR (r) as reported in [19], microRNA/protein degradation of those key molecules, mutual inhibition between miR-451 and AMPK complex and inhibition of mTOR activity by AMPK complex.

Based on these observations, we write the phenomenological equations for the rate change of those key modules (m, a, r) as follows:

$$\frac{dm}{dt} = f(g) + \frac{\Lambda_1 \Lambda_2^2}{\Lambda_2^2 + \Lambda_5 F(a)} - \mu_1 m, \tag{1}$$

$$\frac{da}{dt} = h_1(s_1) + \frac{\Lambda_3 \Lambda_4^2}{\Lambda_4^2 + \Lambda_6 H_1(m)} - \mu_2 a, \tag{2}$$

$$\frac{dr}{dt} = h_2(s_2) + \frac{\Lambda_7 \Lambda_8^2}{\Lambda_8^2 + \Lambda_9 H_2(a)} - \mu_3 r, \tag{3}$$

where g represents the signaling pathways from glucose to miR-451, s_1, s_2 are the signalling pathways to AMPK complex and mTOR, respectively. $\Lambda_1, \Lambda_3, \Lambda_7$ are the autocatalytic enhancement parameters for miR-451, AMPK complex, mTOR, respectively. $\Lambda_2, \Lambda_4, \Lambda_8$ are the Hill-type inhibition saturation parameters from the counterpart of miR-451, AMPK complex, mTOR, respectively. Λ_5 is the inhibition strength of miR-451 by the AMPK complex, Λ_6 is the inhibition strength of the AMPK complex by miR-451, Λ_9 is the inhibition strength of the mTOR by the AMPK complex. Finally μ_1, μ_2, μ_3 are microRNA/protein degradation rates of miR-451, AMPK complex, and mTOR, respectively.

As indicated in equation (1), the signal g increases the rate of miR-451 activation through the function $f(g)$, while AMPK-dependent inhibition of miR-451 is through the function $F(a)$ in the denominator. A requirement of these functions is that $\frac{\partial f}{\partial g} > 0$ for all non-negative g, and $\frac{\partial F}{\partial a} > 0$ for all non-negative a. Similarly, the first term $h_1(s_1)$ on the right-hand side of equation (2) represents an increase in AMPK

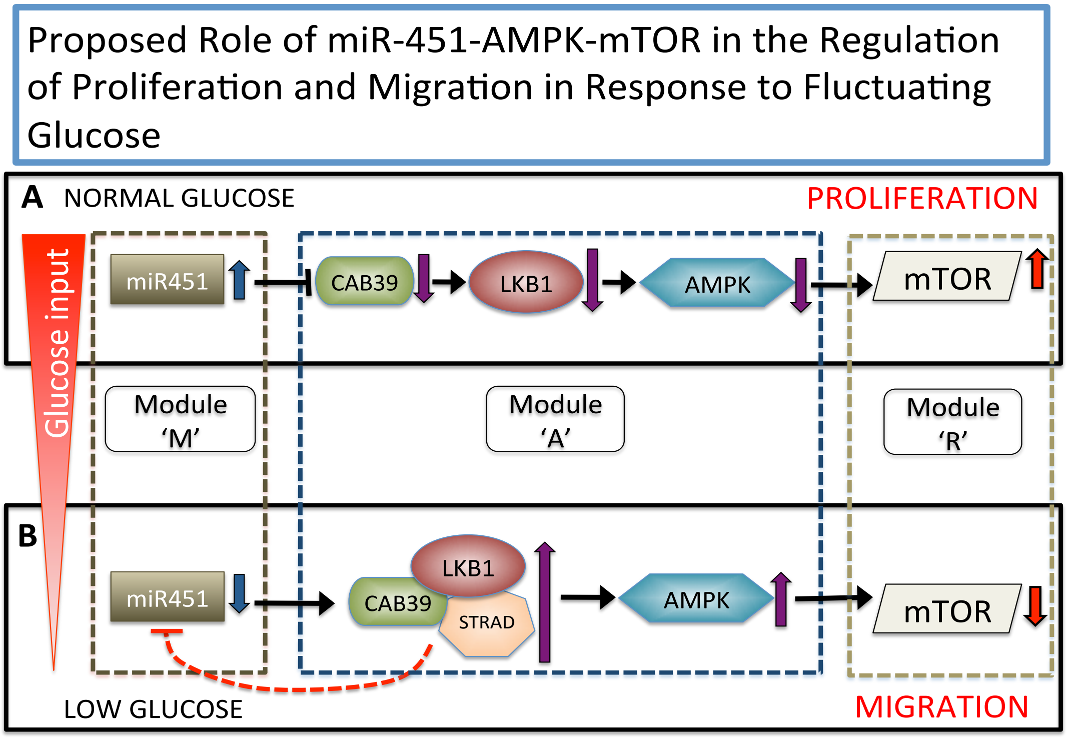

Figure A. Proposed role of miR-451 in the regulation of LKB1/AMPK-mTOR signaling in response to high and low glucose levels [18]. miR-451 levels determine cell migration or proliferation in response to glucose (red triangle on the left) via the AMPK-mTOR network [19]. (A) Normal glucose levels up-regulate miR-451, which leads to down-regulation of the AMPK complex (CAB39/LKB1/AMPK). In turn, up-regulated AMPK levels induce up-regulation of mTOR, which leads to increased proliferation and decreased cell migration. (B) Low glucose levels reduce miR-451 levels, resulting in up-regulation of AMPK activity and down-regulation of mTOR. This leads to reduced cell proliferation and enhanced cell motility. Schematic components of miR-451, CAB39/LKB1/AMPK complex, and mTOR are represented by modules 'M' (box with brown dotted line), 'A' (box with blue dotted line), and 'R' (box with brown dotted line on the right) in our theoretical framework.

activity induced by the signal s, and miR-451-dependent suppression of AMPK activity through the function $H_1(m)$ in the denominator. In the same manner, the first term $h_2(s_2)$ on the right-hand side of equation (3) represents an increase in mTOR activity induced by the signal s_2, and AMPK-dependent suppression of mTOR activity through the function $H_2(a)$ in the denominator. One must also have $\frac{\partial h_1}{\partial s_1} > 0, \frac{\partial h_2}{\partial s_2} > 0, \frac{\partial H_1}{\partial m} > 0, \frac{\partial H_2}{\partial a} > 0$ for all non-negative s_1, s_2, m, a. Based on biological observations (Figure A), we assume that

$$f(g) = \Lambda_g g,\ F(a) = a^2,\ h_1(s_1) = s_1,\ H_1(m) = m^2,\ h_2(s_2) = s_2,\ H_2(a) = a^2. \tag{4}$$

The governing equations (1)-(3) with the simple assumption (4) in a dimensional form become

$$\frac{dm}{dt} = \Lambda_g g + \frac{\Lambda_1 \Lambda_2^2}{\Lambda_2^2 + \Lambda_5 a^2} - \mu_1 m, \tag{5}$$

$$\frac{da}{dt} = s_1 + \frac{\Lambda_3 \Lambda_4^2}{\Lambda_4^2 + \Lambda_6 m^2} - \mu_2 a. \tag{6}$$

$$\frac{dr}{dt} = s_2 + \frac{\Lambda_7 \Lambda_8^2}{\Lambda_8^2 + \Lambda_9 a^2} - \mu_3 r, \tag{7}$$

where all parameters $(\Lambda_G, g, s_1, s_2, \Lambda_i\ (i = 1, \ldots, 9), \mu_i\ (i = 1, 2, 3))$ are non-negative constants.

2 Detailed Mechanics Scheme

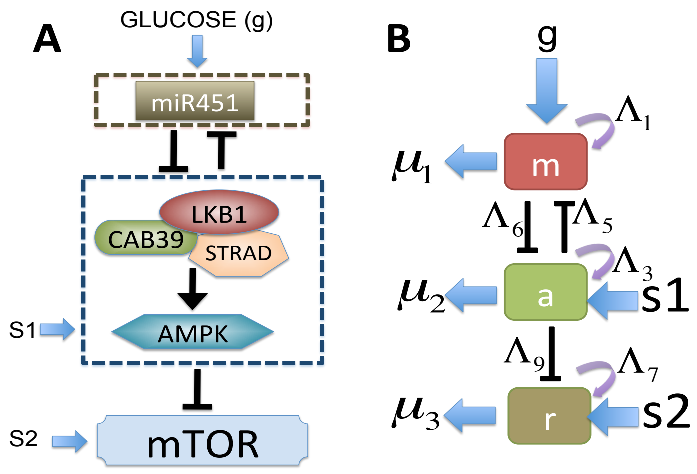

Figure B. A schematic of the core control model. (A) Conceptual model of regulation of miR451, AMPK complex, and mTOR in GBM cell migration and proliferation [19]. (B) Cartoon model (extended from [18]): miR-451 level and activity of its target complex (CAB39/LKB1/AMPK), and mTOR levels were represented by 'm', 'a', and 'r', respectively.

2.1 Mechanical effects on tumor growth: The cell-based component

Cell-mechanics is believed to play a large role in tumor growth and invasion. Mechanical stresses and the signaling pathways influence growth in a phenomenologically-specified manner in our model. We begin in the next section with a description of the cell-based component of the hybrid model.

2.1.1 The forces acting on individual cells

The mechanical behavior of individual cells is based on the model developed by Dallon and Othmer [1] (hereafter the paper and model is denoted DO) and Kim *et al.* [2] (hereafter the paper and model is denoted KSO). The new aspect that is needed in the present context is the core control system (miR-451-AMPK-mTOR) for cell proliferation and migration. The forces on a cell in the DO model include (i) the active forces exerted on neighboring cells or the substrate, (ii) the dynamic drag forces that arise as a moving cell forms and breaks adhesive bonds with neighboring cells, (iii) a static frictional force that exists when cells are rigidly attached to each other or to the substrate, and (iv) a reactive force due to forces exerted by other cells on it. The active force on cell i is denoted $\mathbf{T}_{i,j}$, wherein $j = 0$ denotes the substrate, and the reaction force to this is denoted $\mathbf{M}_{j,i}$. In the context of glioma migration via miR-451-AMPK-mTOR regulation, the specific form of active forces will be introduced in Section 2.1.3. The static force, which is denoted $\mathbf{S}_{j,i}$, is the binding force on the ith cell when bound to the jth. Since $\mathbf{S}_{i,j} = -\mathbf{S}_{j,i}$, the cell-cell forces cancel on all but those cells attached to the substrate. A more detailed discussion of all forces involved can be found in [1, 2]. The total force on the ith cell is then summarized by

$$\mathbf{F}_i = \sum_{j\in\mathcal{N}_i^a} \mathbf{M}_{j,i} + \sum_{j\in\mathcal{N}_i^a} \mathbf{T}_{j,i} + \sum_{j\in\mathcal{N}_i^d} \mu_{ij}(\mathbf{v}_j - \mathbf{v}_i) + \sum_{j\in\mathcal{N}_i^s} \mathbf{S}_{j,i} \tag{8}$$

where $\mathcal{N}_i^a$ denotes the neighbors of i, including the substrate, upon which it can exert traction, $\mathcal{N}_i^d$ is the set of cells (which includes substrate and extracellular matrix) that interact with i via a frictional force, and $\mathcal{N}_i^s$ denotes the set of cells that statically bind to cell i.

2.1.2 Cell growth and the rheology of the cytoplasm

There are two different kinds of cells involved in the system: proliferative cells and motile cells. Proliferative cells are modeled as in the KSO model [2] with intracellular dynamics (miR-451-AMPK-mTOR module) and an algorithm for migratory cells is introduced as in [1, 3]. The KSO model, which is based on an earlier model for the mechanical behavior of cells and tissues under stress [1], addresses four major aspects (i) how an individual cell reacts to forces on it, (ii) how stress affects growth and inhibits growth, (iii) how cells interact mechanically with their surrounding microenvironment, and (iv) how growth and division can be described.

The cells are treated as oriented ellipsoids and their cytoplasm considered as an incompressible, viscoelastic solid. In the absence of growth their volume is preserved (constant under all deformations), and growth is included in series with the active response and the passive forces. Changes in the length of an axis $\mathbf{a}$ of a cell consist of the passive change due to the length of the viscoelastic component (spring-dashpot module; u_a^0) and the change due to the growth (u_a^g) in response to given mechanical forces and biochemical signals (oxygen and glucose). The governing equations of the length of the i-th axis, $i = \mathbf{a}, \mathbf{b}, \mathbf{c}$, of a cell are

$$u_i = u_i^0 + u_i^g, \tag{9}$$

$$\frac{du_i^0}{dt} = \left(\frac{k_i}{\mu_i}[f_i(t) + \bar{p} - f_2(u_i^0)] + \frac{df_i}{dt}\right) \times \left(f_2'(u_i^0) + k_i\right)^{-1}, \tag{10}$$

where u_i is the change in the length of the ith axis, u_i^0 and u_i^g are the changes in the length of the ith axis due to a change in the passive and growth element, respectively, f_i is the magnitude of the force applied at each end, f_2 is the nonlinear spring force from the spring in parallel, k_i is the spring constant for the spring in the Maxwell element, μ_i is the viscous coefficient of the dashpot, $\bar{p}$ is the force due to pressure. See [1] for specific form of the function f_2 and details of how these equations are established. It

is assumed that the passive response is incompressible [2,4], leading to the volume constraint for u_a^0, u_b^0, and u_c^0:

$$\left(\frac{du_a^0}{dt}\right)(u_b^0 + b_0^*)(u_c^0 + c_0^*) + (u_a^0 + a_0^*)\left(\frac{du_b^0}{dt}\right)(u_c^0 + c_0^*) + (u_a^0 + a_0^*)(u_b^0 + b_0^*)\left(\frac{du_c^0}{dt}\right) = 0, \tag{11}$$

where a_0^*, b_0^* and c_0^* are the lengths of three axes $\mathbf{a}, \mathbf{b}, \mathbf{c}$ after growth ($a_0^* = a_0 + u_a^g, b_0^* = b_0 + u_b^g, c_0^* = c_0 + u_c^g$ where a_0, b_0 and c_0 are the initial lengths of three axes). Specific form of the growth term $\dot{u}_i^g$ is introduced below. In the KSO model [2] a cell can grow and divide into two daughter cells following the developed algorithm. This schematic algorithm incorporates the following major aspects (i) how to define an intrinsic cell-cycle time τ_c; (ii) how to define an intrinsic cell volume V_0 that cells attain immediately after division; (iii) how a cell divides under a given stress; (iv) how mechanical stress can inhibit cell growth. A cell relaxes to a spherical shape, whatever their initial shape, in the absence of external forces. In the present work we need to refine our assumptions on growth. In a previous work, Kim *et al.* [2,4] assumed that, in the absence of nutrient limitation, mechanical stress limitations [2,4], or other anti-growth signaling factors [4] cells grow to the volume $2V_0$ and then instantly divide into equal two daughter cells. The orientation of cell division is determined by the direction of the net force exerted on the cell as in [2]. Several control mechanisms may delay or inhibit growth or cell cycle. Many different biomechanical and biochemical factors may affect the growth of normal cells and its regulation in cancerous cells may be very complex. (See the detailed discussion for regulation of cell growth below.)

In the current model, we assume that the control mechanism of growth inhibition has three components. The first component controls the growth along each axis, which is zero when the force acting on the cell in that direction is too compressive or tensile. The second one is switch-like component, which is specific to the model used here, miR-451-AMPK-mTOR-dependent module. Finally, the third control component consists in cell cycle arrest and apoptosis due to chemotherapeutic that target either G_1- or S-G_2-M-phase in a cell cycle. We assume that the growth rate of proliferative cells depends on biomechanical signals (the mechanical stress acting on the cells) and biochemical signals (the up- or down-regulation of miR-451, AMPK, mTOR in the core control system in response to glucose) independently, and relate the growth of the axes of the cell to the tensions along major three axes. Thus we use the multiplicative form [3–5] of the growth rate function for the i-th axis given by

$$\frac{du_i^g}{dt} = f(\sigma) \cdot P(M, A, R, [G_0]) \tag{12}$$

where σ is the force acting on the cell and P is a function of the levels of miR-451 (M), AMPK complex (A), and mTOR (R), and quiescent status ($[G_0]$) in the cell cycle. See Figure C. The growth function $f(\sigma)$ is defined so that cells do not grow if forces are too large, but can grow under sufficiently small tensile and compressive forces. We also assume that the growth rate due to stress decays linearly with increasing compressive or tensile stress. Thus we define $f(\cdot)$ as follows (same as KSO with $\alpha = 0$ there):

$$f(\sigma) = \begin{cases} c^-(\sigma - \sigma^-) & \text{if } \sigma^- \le \sigma < 0, \\ -c^+(\sigma - \sigma^+) & \text{if } 0 \le \sigma \le \sigma^+, \\ 0 & \text{if } \sigma > \sigma^+, \sigma < \sigma^-, \end{cases} \tag{13}$$

where c^+, c^- are positive constants, $\sigma^+ > 0,\ \sigma^- < 0,\ [\sigma^-, \sigma^+]$ is the interval of positive growth, $c^+\sigma^+ = -c^-\sigma^-$. As a result growth is either on or off in the context of mechanical stress acting on the cell. See Figure 4 in [2] for more detail. Cell proliferation depends on levels of many intracellular variables that control internal biological clocks of cell proliferation and migration. In the present work, we assume that the core control system and conditions of G_0 phase ($[G_0] = 0$ or 1) in the cell cycle module also determine cell proliferation, *i.e.,* when the cell i is getting the right proliferation signal from the core

control system (miR-451-AMPK-mTOR) and the cell is in the regular cell-cycle ($[G_0] = 0$). Thus, the function $P(M, A, R, [G_0])$ in the equation (12) is defined as

$$P(M, A, R, [G_0]) = \begin{cases} 1 & \text{if } M > th_M, A < th_A, R > th_R, [G_0] = 0 \\ 0 & \text{otherwise} \end{cases} \tag{14}$$

where M, A, R is the levels of miR-451, AMPK complex, and mTOR, respectively, at the cell site and th_M, th_A, th_R are the threshold values of M, A, R, respectively. See Figure C(B).

Many factors including temporal changes [6] in chemicals or signaling levels, spatial differences [6, 7], growth factors [8], or mechanical stress [8] can affect cell growth even in a simple system such as that in flies. Mechanical stresses can be processed through integrins-cytoskeleton and affect biochemical interactions [8–13], gene expression and overall growth [14, 15]. Both compression and tension [8, 13–15] may promote cell division through the formation of mitotic spindles along the main axis of tension. This phenomenon of growth inhibition under mechanical constraints was also modeled with a simplification of those complicated relationships between growth and stresses in the context of growth of multilayer spheroids [2, 4, 16] and compared to experiments [14, 15]. Mechanical interaction between a cell and substrate was also shown to govern growth behavior in epithelial cell growth [17]. The same hybrid model approach was proven to be useful in reproducing growth patterns of tumor epithelial cells under mechanical stresses in ductal carcinoma DCIS *in situ* [4, 5].

2.1.3 Active force and equations of motion

For the force balance equations for each cell we take into consideration all the forces acting on the cell: the active forces, adhesion forces, internal pressure, and other forces as discussed in Section 2.1.1. We first describe the active force here. In DO model, the active force is generated by pulling on the neighboring cell whose center is closest to the line in the direction of desired motion when the cell is not in contact with the substrate. In the current model, we assume that migratory cells transmit the active force directly to the substrate and generate the active force by pulling the substrate. The model can easily be extended to incorporate active forces generated by all cells, but the cells in the interior of an aggregate need to connect to the surrounding tissue in order to do meaningful work toward moving the aggregate [1]. Furthermore, as we mentioned earlier, glioma cells in the interior of the growing tumor mass may grow but do not migrate. We assume that a glioma cell becomes motile (which is the case when the AMPK level stays above the threshold value ($A > th_A$)), this generates an active force in the direction of a gradient consisting of a combination of glucose and chemoattractants, with a probability as well as in the direction from random motion as long as it is not surrounded by neighboring cells. The active force $\mathbf{T}_i$ for migratory cell i is given by

$$\mathbf{T}_i = \phi(A)\left(\psi_1 \mathbf{d}_r + \psi_2 \frac{\nabla G}{\sqrt{K_G + |\nabla G|^2}} + \psi_3 \frac{\nabla C}{\sqrt{K_C + |\nabla C|^2}}\right) \tag{15}$$

where $\mathbf{d}_r$ is a unit vector of the moving direction from random motion, G, C are the concentrations of glucose and a chemoattractant, respectively (described in Materials and Methods Section in the main text), ψ_1, ψ_2, ψ_3 are scaling factors of weight distribution favoring random motion, glucose and other chemoattractants, respectively ($\psi_1, \psi_2, \psi_3 \in [0, 1]$; $\psi_1 + \psi_2 + \psi_3 = 1$), A is the level of AMPK complex at the ith cell site. We allow a small randomness in the magnitude of active force. Then, the indicator function $\phi(A)$ is given by

$$\phi(A) = \begin{cases} F_0 \phi_r & \text{if } A_i > th_A \text{ and the cell does not have physical constraints} \\ 0 & \text{otherwise,} \end{cases} \tag{16}$$

where F_0 is the basal magnitude of the active force ($0 \le |\mathbf{T}_i| \le F_0$) and ϕ_r is a random number ($\phi_r \in [0.8, 1.2]$). Therefore, the active force is completely turned off for cells in the proliferative phase

$(M > th_A, A < th_A, R > th_R)$ or cells under physical constraints. For example, when a cell is completely surrounded by neighboring cells, the active force is also turned off. From the formulation in the equation (15), it would be turned off when gradients of glucose (∇G) and chemoattractants (∇C) are zero in the absence of random motility ($\psi_1 = 0$), *i.e.,* no active force is generated in the absence of chemotactic signals.

In summary, Newton's law for the ith cell reduces to

$$A_{if}\mu_f \mathbf{v}_i + A_{is}\mu_s \mathbf{v}_i + \mu_{cell}\sum_{j\in\mathcal{N}_i} A_{ij}(\mathbf{v}_i - \mathbf{v}_j) + \\ + \frac{A}{6\pi r_{ib}}\left(\mathbf{T}_i + \mathbf{R}^*_{0,i} + \sum_{j\in\mathcal{N}_i}\mathbf{A}_{i,j} + \sum_{j\in\mathcal{N}_i}\mathbf{R}_{j,i} + \sum_{j\in\mathcal{N}_i}\mathbf{R}^*_{j,i}\right) = 0, \quad (17)$$

where $\mathbf{v}_i$ is the velocity of cell i, $\mathcal{N}_i$ is the neighborhood of cell i, μ_{cell} (resp., μ_s, μ_f) is the degree of adhesiveness between the cells (resp., between the substrate and the cells, and the fluid viscosity), and $r_{ib} = u_b + b_0$. $A_{ij} = A_{ij}(t)$, $A_{if} = A_{if}(t)$, A_{is} are the areas of contact regions between cell i and cell j, cell i and the interstitial fluid or matrix, and cell i and the substrate at time t respectively. $A = A(t)$ is the total area of an undeformed cell. The solution of this equation (17) provides a trajectory of cell i. For more details see DO. Parameters in the cell-based component are listed in Table 2 in the main text.

These mechanical components of the cell-based model, equations (9)-(17), are integrated with the reaction diffusion equations and the core control system in the hybrid framework in the main text. Figure D shows the overall interactions and flow of three major model components: miR-451-AMPK-mTOR system at the intracellular level, cell mechanics at the cellular level and microenvironmental factors such as glucose and oxygen.

3 Parameter estimation

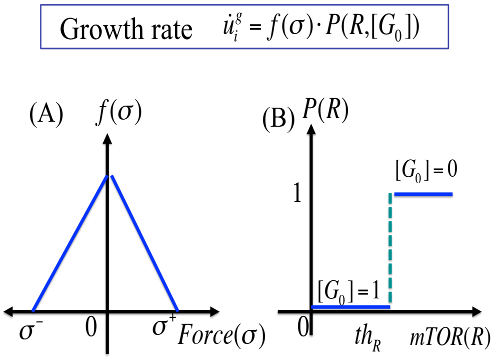

Figure C. Growth rate $\frac{du_i^g}{dt} = f(\sigma)P(R,[G_0])$ as a function of force along the i-th axis (σ), mTOR level (R) and $[G_0]$ at the cell site.

3.1 Tumor module

We estimate some of parameters and reference values in the following.

Parameters:

(i) D_K (Diffusion coefficient of oxygen): 2.15×10^{-5} cm^2/s [2,20], 1.82×10^{-5} cm^2/s [2,21] 2.0×10^{-5} cm^2/s [22,23]. We take $D_K = 2.0 \times 10^{-5}$ cm^2/s as in [22,23].

(ii) D_G (Diffusion coefficients of glucose): Diffusion coefficients of glucose were measured to be 6.7×10^{-7} cm^2/s in the brain [24], 1.3×10^{-6} cm^2/s in collagen gel [25], and 1.1×10^{-6} cm^2/s in multi-cellur tumor spheroids [2,26]. Effective diffusion coefficient of glucose in artificial biofilm was 6.46×10^{-6} cm^2/s [2,27]. In order to simulate the tumor growth in the brain, we take $D_G = 6.7 \times 10^{-7}$ cm^2/s as in [24].

(iv) D_C (Diffusion coefficient of chemoattractant): The diffusion coefficient of chemoattractant was taken from one of EGF [28], $D_C = 1.66 \times 10^{-6}$ $cm^2/s.$

(iii) D_P (Diffusion coefficient of MMPs): In experiments of the movement of MMP-1in the collagen fibril, Saffarian *et al.* (2004) [29] estimated the diffusion coefficient of MMPs as $(8.0 \pm 1.5) \times 10^{-9}$ cm^2/s for inactive mutant and $(8.0 \pm 1.5) \times 10^{-9}$ cm^2/s for wild-type activated MMP-1, respectively. For our simulation, we take $D_P = 8.0 \times 10^{-9}$ $cm^2/s.$

(iii) D_D (Diffusion coefficient of chemo-drugs): In a study [23] of effects of cell-cycle heterogeneity on the response of a tumour to chemotherapy, various diffusion coefficients of chemo-drugs were used from [30]: 6.9×10^{-6} cm^2/s (CYC202), 7.6×10^{-6} cm^2/s (Cisplatin), 2.0×10^{-6} cm^2/s (Taxotere), 1.3×10^{-6} cm^2/s (Taxol). We take the diffusion coefficient of CYC202, $D_D = 6.9 \times 10^{-6}$ cm^2/s in this work.

(v) l_c^K (oxygen consumption rate) and μ_K: Powanthil *et al.* [31] took the oxygen consumption rate of 0.2 s^{-1} based on the relation $L = \sqrt{D_K/\phi}$ where ϕ is rate of oxygen consumption by a cell at a cell site in the exponential decay term. In a similar fashion, we find $\mu_K = D_K/L^2 = 2.0\times10^{-5}$ $s^{-1} = 7.2\times10^{-2}$ h^{-1} based on the relationship $L = \sqrt{D_K/\mu_K} = 1cm.$

(v) l_c^G (glucose consumption rate) : The measured level $\alpha = 1.6$ $pg/cell/min$ for piecewise increasing linear consumption term $\alpha(G)n$ was used in a glioma cell migration study [32] with a threshold value $G_1^{th} = 2.0 \times 10^{-4}$ g/cm^3, taken from the work of Li *et al.* [33]. We take $l_c^G = 0.8$ $pg/cell/min$ and use a sphere with a radius $r = 8 - 20$ μm for estimation of cell volume.

(v) l_1 (ECM degradation rate by MMPs): It is hard to measure the decay rate of MMPs due to it's fast decay. Eisenberg *et al.* used the ECM degradation rate, 1.17×10^4 $cm^3g^{-1}s^{-1}$ in a myoferlin-mediated invasion model in breast cancer. We take the adjusted value l_1=3.0×10^4 $cm^3g^{-1}s^{-1}$ from [34].

(vi) : μ_G (overall glucose consumption rate in brain tissue) : Insulin-independent rate constant of tissue glucose uptake was measured to be 0.0226 $min^{-1} = 1.356$ h^{-1} in healthy individuals [35]. We take $\mu_G = 0.0034$ min^{-1}.

(vi) : μ_C (Decay rate of chemoattractant): There exist several factors that may contribute to cell invasion in the brain tissue: Extra cellular matrix (ECM) and MMPs [36–38], chemotactic attractants (EGF family [39]; the TGF-β family [40]; scatter factor/hepatocyte growth factor (SF/HGF) [41]; SDF-1 [42]; certain lipids [43]), even other cell types such as microglia through indirect stimulation [44] . It is not clear which combination of those would generate the best outcomes. Here we take the decay rate of EGF for decay rate of chemoattractants: $\mu_C = 8.02 \times 10^{-6}$ s^{-1} [45].

(iii) μ_D (Decay rate of chemo-drugs): In a study [23] of effects of cell-cycle heterogeneity on the response of a tumour to chemotherapy, various decay rates of chemo-drugs were used from [30]: 1.849 h^{-1} (CYC202), 1.316 h^{-1} (Cisplatin), 0.05634 h^{-1} (Taxotere), 0.05776 h^{-1} (Taxol). We take the diffusion coefficient of CYC202, $mu_D = 1.849$ h^{-1} in this work.

Reference values:

(i) G^*: Sander and Deisboeck [32] used the characteristic concentration 2×10^{-4} g/cm^3 of glucose with high values 6×10^{-4} g/cm^3 in the far field (see also [46,47]). In *in vitro* experimental study by Godlewski

et al. [19], low (0.3 g/l) and high (4.5 g/l) levels of glucose induced the up- and down-regulated expressions of miR-451 and mTOR (down- and up-regulation of AMPK complex), respectively. We take the high glucose level, $G^* = 4.5 \times 10^{-3}$ g/cm^3, as a reference value.

(ii) C^*: EGF is known to be chemoattractants in glioma cell lines [39]. We take the reference value $C^* = 1.0 \times 10^{-8}$ g/cm^3 from experimental studies of the role of EGF in the regulation of cancer progression [48, 49].

(iii) ρ^*: ECM concentration was estimated to be 0.5-2.0 mg/ml in an experimental study of growth/invasion patterns of glioma spheroids. Stein *et al.* [50] studied growth patterns of glioma cell lines, U87 (wild type) and U87ΔEGFR (its mutant), embedded in collagen I (major ECM component) of concentration of 2.6 mg/ml. We take $\rho^* = 1.0 \times 10^{-3}$ g/cm^3.

(iv) P^*: MMP concentrations were measured to be 1.6×10^{-9} g/cm^3 in a breast cancer cell invasion study [51]. On the other hand, it was observed that PCK3145 may down-regulate MMP-9 expression in prostate cancer patients with up-regulated MMP-9 level of $> 1.0 \times 10^{-7} g/cm^3$ [52]. We take the bit high levels of MMPs: $P^* = 1.0 \times 10^{-7}$ g/cm^3.

Table 3 in the main text lists reference values and all parameter values above.

3.2 Core control system (miR-451, AMPK, mTOR)

miRNAs are typically more stable than their targets [53, 54] and the typical half-life of a miRNA is much larger than one of AMPK [55, 56] and mTOR, leading to the small values of the fractions $\epsilon_1 = \frac{\mu_1}{\mu_2} = 0.02$, $\epsilon_2 = \frac{\mu_1}{\mu_3} = 0.02$ [3, 18, 57]. From the estimated concentrations of miRNAs (80 pM – 2.2 μM) in an animal cell (assuming 1,000-25,000 μm^3 volume) [58], we take $m^* = 1.0$ μM. The normal (high; 4.5 g/l) and low (0.3 g/l) glucose levels were used in [19] in order to test the effect of glucose on the miR451 expression and AMPK activities, and proliferation/migration patterns of glioma cells. Using this range of glucose levels ($0.3 - 4.5$ g/l) in [19] and the miR-451 reference value (m^*), we determined the glucose signaling strength, $(2.4 \times 10^{-5} - 2.4 \times 10^{-3})$ $\mu M/h$, resulting in a range of dimensionless glucose input level $\lambda_g G = \frac{\Lambda_g g}{\mu_1 m^*} = 0.01 - 1.0$. Using the reference value of AMPK, $a^* = 100 nM$ taken from the measured values (35-150 nM) in rat liver [59], and taking the signal source of the AMPK complex, $s_1 = 2.4$ nM/h, we estimate the dimensionless value of signaling strength to the AMPK complex to be $S_1 = \frac{s_1}{\mu_2 a^*} = 0.2$ [3, 18]. By observing the same patterns of up- and down-regulation of mTOR as ones of miR-451 in response to various glucose levels, we take slightly larger values of $S_2 = \frac{s_2}{\mu_3 r^*} = 1.2$ in the work. We take the dimensionless parameter value $\lambda_1 = \frac{\Lambda_1}{\mu_1 m^*} = 4.0$ by assuming that the autocatalytic rate (Λ_1) of miR-451 is at least 4-fold larger than its negative contribution ($\mu_1 m^*$) from its decay/consumption in the absence of the inhibitory pathway from the AMPK complex [3, 18]. Relying on the observed mutual antagonism between miR-451 and AMPK complex, we also take $\lambda_3 = \frac{\Lambda_3}{\mu_2 a^*} = 4.0$ in a similar fashion. The Hill-type coefficients $\Lambda_2, \Lambda_4, \Lambda_6$ (and their corresponding dimensionless parameters $\lambda_2, \lambda_4, \lambda_6$) are fixed to be unity [3, 18]. Once the parameters above determined, we fitted the data in the level of LKB1/AMPK activity in [19] in response to negative control and over expressed levels of miR-451 (Fig 5B in [19]) in the following way: The steady state of AMPK complex (A^s) in terms of miR-451 level (M^s) and other parameters in the miR-451-AMPK model can be rewritten by

$$A^s = S_1 + \frac{\lambda_3 \lambda_4^2}{\lambda_4^2 + \beta (M^s)^2} = 0.2 + \frac{4}{1 + \beta (M^s)^2}. \tag{18}$$

The up-regulated AMPK complex level (∼500 *pmol* of phosphate incorporated in a dimensional form) for negative control of miR-451 was down-regulated (∼100 *pmol* of phosphate incorporated in a dimensional form). Using equation (18) above, we estimated the parameter value of β to be 1.0 which gives the low AMPK complex activity ($\sim A^s = 0.9$) in response to a high dimensionless miR-451 level ($M^s = 4.2$)

and the high AMPK complex activity ($A^s = 4.2$) in response to negative control of the miR-451 level ($M^s = 0.0$), resulting in a reasonable ∼4.7-fold difference in the AMPK complex activities as in the experiments in [19]. Finally, by observing the behavior of the system and experimental data, we assume that the inhibition strength ($\alpha = 1.6$) of miR-451 by the AMPK complex is bit larger than the inhibition strength ($\beta = 1.0$) of the AMPK complex by miR-451 [3, 18] while keeping the same value for the inhibition strength ($\gamma = 1.0$) of mTOR by the AMPK complex.

4 Nondimensionalization

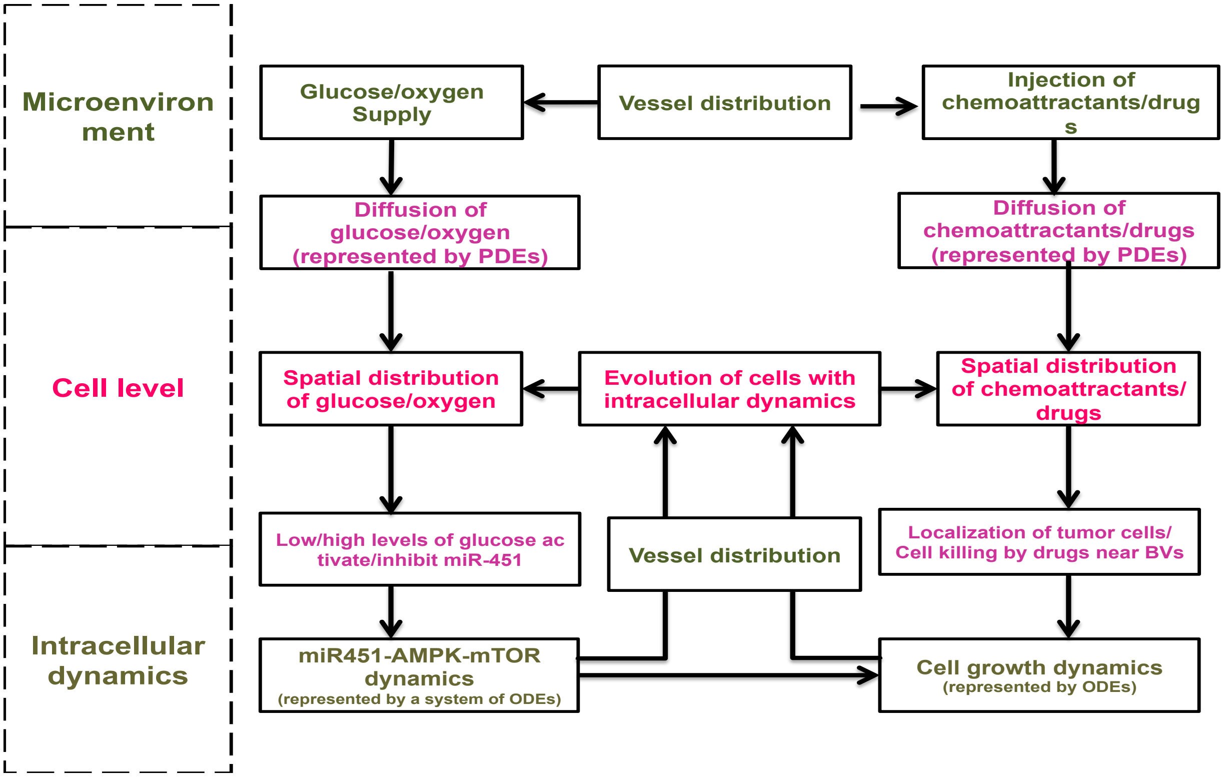

Figure D. Schematic diagram of the model showing the appropriate scales involved.

The characteristic distance is the 1 cm, $L = 1$ cm, and we use $T = 1$ h to get dimensionless variables and parameters:

$$\begin{aligned}
&\bar{t} = \frac{t}{T},\ \bar{x} = \frac{x}{L},\ \bar{K} = \frac{K}{K^*},\ \bar{G} = \frac{G}{G^*},\ \bar{C} = \frac{C}{C^*},\ \bar{\rho} = \frac{\rho}{\rho^*},\ \bar{P} = \frac{P}{P^*},\ \bar{D} = \frac{D}{D^*},\ \bar{D}_K = \frac{T}{L^2}D_K,\\
&\bar{D}_G = \frac{T}{L^2}D_G,\ \bar{D}_C = \frac{T}{L^2}D_C,\ \bar{D}_P = \frac{T}{L^2}D_P,\ \bar{D}_D = \frac{T}{L^2}D_D,\ \bar{r}_K = \frac{T}{K^*}r_K,\\
&\bar{l}_c^K = \frac{T}{K^*}l_c^K,\ \bar{r}_G = \frac{T}{G^*}r_G,\ \bar{l}_c^G = \frac{T}{G^*}l_c^G,\ \bar{l}_{in}^C = \frac{T}{C^*}l_{in}^C,\ \bar{l}_1 = Tl_1P^*,\ \bar{l}_2 = T\ l_2,\\
&\bar{\rho}_* = \frac{\rho_*}{\rho^*},\ \bar{l}_3 = Tl_3,\ \bar{\mu}_G = T\mu_G,\ \bar{\mu}_C = T\mu_C,\ \bar{\mu}_P = T\mu_P.
\end{aligned} \tag{19}$$

The following dimensionalization was performed to get the dimensionless key control equations in the main section

$$
\begin{aligned}
&T = \mu_1 t,\ M = \frac{m}{m^*},\ A = \frac{a}{a^*},\ G = \frac{g}{m^*},\ \lambda_g = \frac{\Lambda_g}{\mu_1},\ S_1 = \frac{s_1}{\mu_2 a^*},\ S_2 = \frac{s_2}{\mu_3 r^*},\\
&\lambda_1 = \frac{\Lambda_1}{\mu_1 m^*},\ \lambda_2 = \Lambda_2,\ \lambda_3 = \frac{\Lambda_3}{\mu_2 a^*},\ \lambda_4 = \Lambda_4,\ \lambda_5 = \frac{\Lambda_7}{\mu_3 r^*},\ \lambda_6 = \Lambda_8,\ \alpha = \Lambda_5 (a^*)^2,\\
&\beta = \Lambda_6 (m^*)^2,\ \gamma = \Lambda_9 (a^*)^2,\ \epsilon_1 = \frac{\mu_1}{\mu_2},\ \epsilon_2 = \frac{\mu_1}{\mu_3}.
\end{aligned}
\tag{20}
$$

5 Dynamics of the system

Figure E shows spatial profiles of concentrations of oxygen ($K(\mathbf{x}, t)$ in (A)), glucose ($G(\mathbf{x}, t)$ in (B)), ECM ($\rho(\mathbf{x}, t)$ in (C)), and MMP ($P(\mathbf{x}, t)$ in (D)) at corresponding times ($t = 0, 80, 160, 240\ h$) as that of the spatial patterns of the tumour cells that are given in Figures 5A-5D in the main text. While invasive glioma cells after surgery undergo random motion ($\psi_1 = 0.6$) in the brain tissue, they are also attracted to BV sites via biochemical signals, in this case glucose and oxygen levels, which have highest values at fixed source points (BV sites; Figure E(A),E(B)). Active force is generated by the migratory cells when the cell receives migratory signals and these motile cells move forward by secreting MMPs (Figure E(D)), thus degrading ECM (Figure E(C)).

References

1. Dallon JC, Othmer HG (2004) How cellular movement determines the collective force generated by the dictyostelium discoideum slug. J Theor Biol 231: 203–222.

2. Kim Y, Stolarska M, Othmer H (2007) A hybrid model for tumor spheroid growth in vitro I: Theoretical development and early results. Math Models Methods in Appl Scis 17: 1773-1798.

3. Kim Y, Roh S (2013) A hybrid model for cell proliferation and migration in glioblastoma. Discrete and Continuous Dynamical Systems-B 18: 969–1015.

4. Kim Y, Stolarska M, Othmer H (2011) The role of the microenvironment in tumor growth and invasion. Prog Biophys Mol Biol 106: 353-379.

5. Kim Y, Othmer H (2013) A hybrid model of tumor-stromal interactions in breast cancer. Bull Math Biol 75: 1304–1350.

6. Wartlick O, Mumcu P, Kicheva A, Bittig T, Seum C, et al. (2011) Dynamics of DPP signaling and proliferation control. Science 331: 1154–1159.

7. Day S, Lawrence P (2000) Measuring dimensions: the regulation of size and shape. Development 127: 2977–2987.

8. Wartlick O, Mumcu P, Julicher F, Gonzalez-Gaitan M (2011) Understanding morphogenetic growth control - lessons from flies. Nat Rev Mol Cell Biol 12: 594–604.

9. Park J, Kwak H, Lee S (2008) Role of hyaluronan in glioma invasion. Cell Adhesion and Migration 2: 202-207.

10. Gotte M, Yip G (2006) Heparanase, hyaluronan, and CD44 in cancers: a breast carcinoma perspective. Cancer Research 66: 10233–10237.

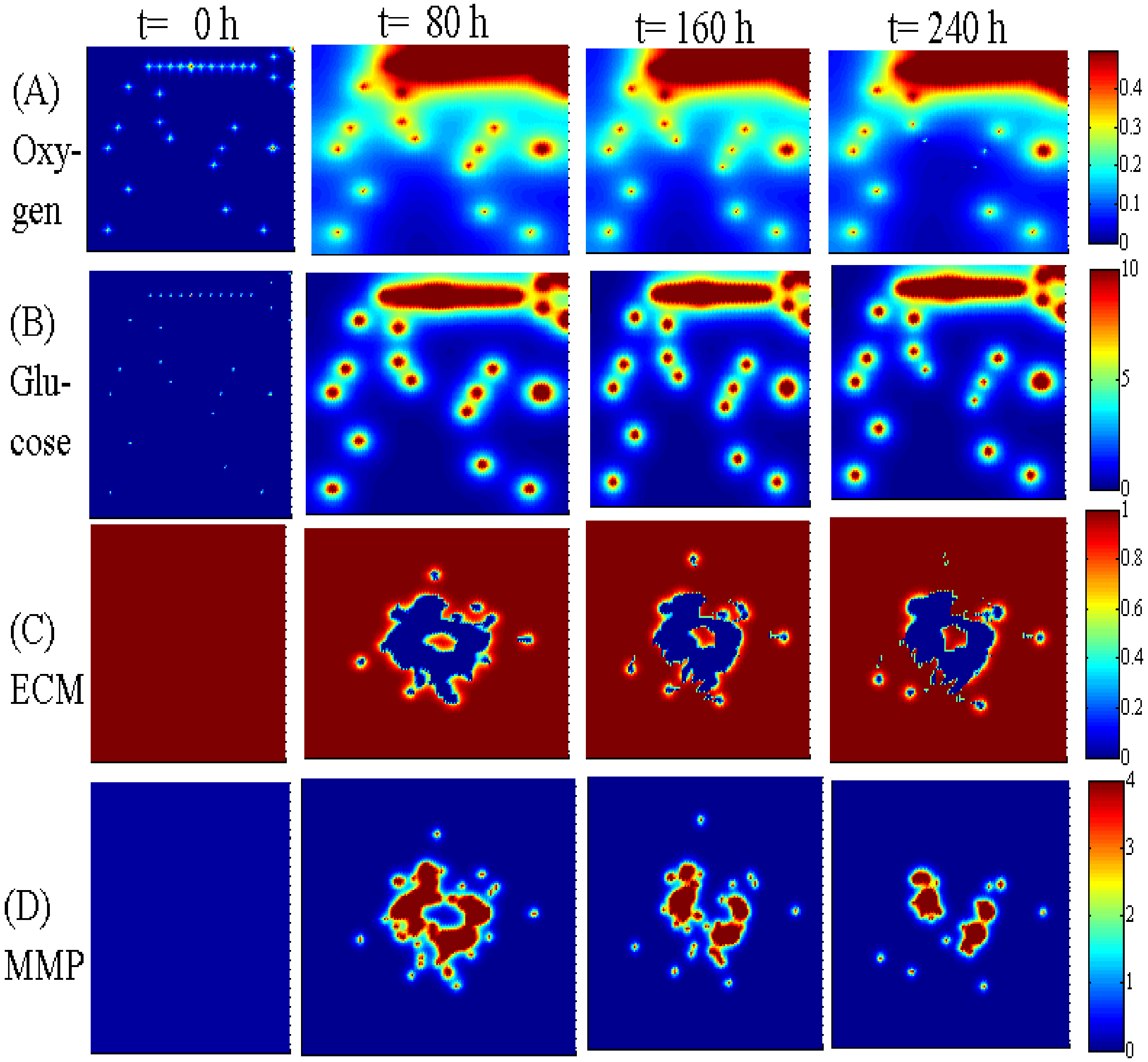

Figure E. Spatial profiles of concentrations of diffusive molecules in Figures 5A-5D in the main text: oxygen ($K(\mathbf{x}, t)$ in (A)), glucose ($G(\mathbf{x}, t)$ in (B)), ECM ($\rho(\mathbf{x}, t)$ in (C)), and MMP ($P(\mathbf{x}, t)$ in (D)) at the corresponding time ($t = 0, 80, 160, 240$ h).

11. Cho A (2004) Life's patterns: No need to spell it out? Science 303: 782–783.

12. Shraiman B (2005) Mechanicall feedback as a possible regulator of tissue growth. PNAS 102: 3318-3323.

13. Koike C, McKee T, Pluen A, Ramanujan S, Burton K, et al. (2002) Solid stress facilitates spheroid formation: potential involvement of hyaluronan. British Journal of Cancer 86: 947–953.

14. Helmlinger G, Netti P, Lichtenbeld H, Melder R, Jain R (1997) Solid stress inhibits the growth of multicellular tumor spheroids. Nature Biotechnology 15: 778-783.

15. Cheng G, Tse J, Jain R, Minn L (2009) Micro-environmental mechanical stress controls tumor spheroid size and morphology by suppressing proliferation and inducing apoptosis in cancer cells. PLoS One 4: e4632.

16. Stolarska M, Kim Y, Othmer H (2009) Multiscale models of cell and tissue dynamics. Phil Trans Roy Soc A 367: 3525-3553.

17. Galle J, Loeffler M, Drasdo D (2005) Modeling the effect of deregulated proliferatin and apoptosis on the growth dynamics of epithelial cell populations i vitro. Biophysical J 88: 62-75.

18. Kim Y, Roh S, Lawler S, Friedman A (2011) miR451 and AMPK/MARK mutual antagonism in glioma cells migration and proliferation. PLoS One 6: e28293.

19. Godlewski J, Nowicki M, Bronisz A, Palatini GNJ, Lay MD, et al. (2010) Mircrorna-451 regulates LKB1/AMPK signaling and allows adaptation to metabolic stress in glioma cells. Molecular Cell 37: 620–632.

20. McCabe M, Laurent T (1975) Diffusion of oxygen, nitrogen and water in hyaluronate solutions. Biochimica et Biophysica Acta 399: 131-138.

21. Mueller-Klieser W, Sutherland R (1984) Oxygen consumption and oxygen diffusion properties of multicellular spheroids from two different cell lines. Adv Exp Med Biol 180: 311-321.

22. Owen M, Byrne H, Lewis C (2004) Mathematical modelling of the use of macrophages as vehicles for drug delivery to hypoxic tumour sites. J Theor Biol 226: 377–391.

23. Powathil G, Gordon K, Hill L, Chaplain M (2012) Modelling the effects of cell-cycle heterogeneity on the response of a solid tumour to chemotherapy: Biological insights from a hybrid multiscale cellular automaton model. J Theor Biol 308: 1–19.

24. Jain R (1987) Transport of molecules in the tumor interstitium: a review. Cancer Res 47: 3039-51.

25. Rong Z, Cheema U, Vadgama P (2006) Needle enzyme electrode based glucose diffusive transport measurement in a collagen gel and validation of a simulation model. Analyst 131: 816–821.

26. Casciari J, Sotirchos S, Sutherland R (1988) Glucose diffusivity in multicellular tumor spheroids. Cancer Research 48: 3905-3909.

27. Zhang T, Fang H (2005) Effective diffusion coefficients of glucose in artificial biofilms. Environmental Technology 26: 155-160(6).

28. Thorne R, Hrabetova S, Nicholson C (2004) Diffusion of epidermal growth factor in rat brain extracellular space measured by integrative optical imaging. J Neurophysiol 92: 3471-3481.

29. Saffarian S, Collier I, Marmer B, Elson E, Goldberg G (2004) Interstitial collagenase is a brownian ratchet driven by proteolysis of collagen. Science 306: 108–111.

30. Gordon K (2006) Mathematical modelling of cell-cycle-dependent chemotherapy drugs-implications for cancer treatment. PhD Thesis, University of Dundee, Dundee: 154–202.

31. Powathil G, Kohandel M, Milosevic M, Sivaloganathan S (2012) Modeling the spatial distribution of chronic tumor hypoxia: implications for experimental and clinical studies. Comput Math Methods Med 2012: 410602.

32. Sander L, Deisboeck T (2002) Growth patterns of microscopic brain tumors. Phys Rev E 66: 051901.

33. Li C (1982) The glucose distribution in 9l rat brain multicell tumor spheroids and its effect on cell necrosis. Cancer 50: 2066-2073.

34. Eisenberg M, Kim Y, Li R, Ackerman W, Kniss D, et al. (2011) Modeling the effects of myoferlin on tumor cell invasion. Proc Natl Acad Sci USA 108: 20078–20083.

35. Gaetano A, Arino O (2000) Mathematical modelling of the intravenous glucose tolerance test. J Math Biol 40: 136–168.

36. Choe G, Park J, Jouben-Steele L, Kremen T, Liau L, et al. (2002) Active matrix metalloproteinase 9 expression is associated with primary glioblastoma subtype. Clin Cancer Res 8: 2894-2901.

37. Chintala S, Tonn J, Rao J (1999) Matrix metalloproteinases and their biological function in human gliomas. Int J Dev Neurosci 17: 495-502.

38. Jaalinoja J, Herva R, Korpela M, Hoyhtya M, Turpeenniemi-Hujanen T (2000) Matrix metalloproteinase 2 (MMP-2) immunoreactive protein is associated with poor grade and survival in brain neoplasms. J Neurooncol 46: 81-90.

39. Lund-Johansen M, Bjerkvig R, Humphrey P, Bigner S, Bigner D, et al. (1990) Effect of epidermal growth factor on glioma cell growth, migration, and invasion in vitro. Cancer Res 50: 6039–6044.

40. Platten M, Wick W, Weller M (2001) Malignant glioma biology: role for TGF-beta in growth, motility, angiogenesis, and immune escape. Microsc Res Tech 52: 401-410.

41. Lamszus K, Schmidt N, Jin L, Laterra J, Zagzag D, et al. (1998) Scatter factor promotes motility of human glioma and neuromicrovascular endothelial cells. Int J Cancer 75: 19-28.

42. Zhou Y, Larsen P, Hao C, Yong V (2002) CXCR4 is a major chemokine receptor on glioma cells and mediates their survival. J Biol Chem 277: 49481-49487.

43. Young N, Brocklyn J (2007) Roles of sphingosine-1-phosphate (s1p) receptors in malignant behavior of glioma cells. differential effects of S1P2 on cell migration and invasiveness. Exp Cell Res 313: 1615-1627.

44. Watters J, Schartner J, Badie B (2005) Microglia function in brain tumors. J Neurosci Res 81: 447-455.

45. Kudlow J, Cheung C, Bjorge J (1986) Epidermal growth factor stimulates the synthesis of its own receptor in a human breast cancer cell line. J Biol Chem 261: 4134-4138.

46. Deisboeck T, Berens M, Kansal A, Torquato S, Stemmer-Rachamimov A, et al. (2001) Pattern of self-organization in tumour systems: complex growth dynamics in a novel brain tumour spheroid model. Cell Prolif 34: 115-134.

47. Kim Y, Lawler S, Nowicki M, Chiocca E, Friedman A (2009) A mathematical model of brain tumor : pattern formation of glioma cells outside the tumor spheroid core. J Theo Biol 260: 359-371.

48. Sadlonova A, Novak Z, Johnson M, Bowe D, Gault S, et al. (2005) Breast fibroblasts modulate epithelial cell proliferation in three-dimensional in vitro co-culture. Breast Cancer Res 7: R46-59.

49. Boccardo F, Lunardi G, Petti A, Rubagotti A (2003) Enterolactone in breast cyst fluid: correlation with EGF and breast cancer risk. Breast Cancer Res Treat 79: 17-23.

50. Stein A, Demuth T, Mobley D, Berens M, Sander L (2007) A mathematical model of glioblastoma tumor spheroid invasion in a three-dimensional in vitro experiment. Biophys J 92: 356-365.

51. Jia Y, Zeng Z, Markwart S, Rockwood K, Ignatoski K, et al. (2004) Integrin fibronectin receptors in matrix metalloproteinase-1-dependent invasion by breast cancer and mammary epithelial cells. Cancer Res 64: 8674–8681.

52. Annabi B, Bouzeghrane M, Currie J, Hawkins R, Dulude H, et al. (2005) A PSP94-derived peptide PCK3145 inhibits MMP-9 secretion and triggers cd44 cell surface shedding: implication in tumor metastasis. Clin Exp Metastasis 22: 429–439.

53. Khanin R, Vinciotti V (2008) Computational modeling of post-transcriptional gene regulation by micrornas. J Comput Biol 15: 305-316.

54. Yao G, Lee T, Mori S, Nevins J, You L (2008) A bistable RB-E2F switch underlies the restriction point. Nat Cell Biol 10: 476-482.

55. Gantier M, McCoy C, Rusinova I, Saulep D, Wang D, et al. (2011) Analysis of microRNA turnover in mammalian cells following dicer1 ablation. Nucleic Acids Res 39: 5692-5703.

56. Crute B, Seefeld K, Gamble J, Kemp B, Witters L (1998) Functional domains of the alpha1 catalytic subunit of the AMP-activated protein kinase. J Biol Chem 273: 35347-35354.

57. Aguda B, Kim Y, Hunter M, Friedman A, Marsh C (2008) MicroRNA regulation of a cancer network: Consequences of the feedback loops involving miR-17-92, E2F, and Myc. PNAS 105: 19678-19683.

58. Ragan C, amd MA Ragan MZ (2011) Quantitative prediction of miRNA-mRNA interaction based on equilibrium concentrations. PLoS Comput Biol 7: e1001090.

59. Hardie D, Salt I, Hawley S, Davies S (1999) AMP-activated protein kinase : an ultrasensitive system for monitoring cellular energy charge. Biochem J 338: 717-722.

60. Mercapide J, Cicco R, Castresana J, Klein-Szanto A (2003) Stromelysin-1/matrix metalloproteinase-3 (MMP-3) expression accounts for invasive properties of human astrocytoma cell lines. Int J Cancer 106: 676-682.

61. Kim Y, Friedman A (2010) Interaction of tumor with its microenvironment : A mathematical model. Bull Math Biol 72: 1029-1068.

62. Kaufman L, Brangwynne C, Kasza K, Filippidi E, Gordon V, et al. (2005) Glioma expansion in collagen I matrices: Analyzing collagen concentration-dependent growth and motility patterns. Biophys J BioFAST 89: 635-650.
